# Acid sphingomyelinase inhibition protects mice from lung edema and lethal *Staphylococcus aureus* sepsis

**DOI:** 10.1007/s00109-014-1246-y

**Published:** 2015-01-25

**Authors:** Huiming Peng, Cao Li, Stephanie Kadow, Brian D. Henry, Jörg Steinmann, Katrin Anne Becker, Andrea Riehle, Natalie Beckmann, Barbara Wilker, Pin-Lan Li, Timothy Pritts, Michael J. Edwards, Yang Zhang, Erich Gulbins, Heike Grassmé

**Affiliations:** 1 Department of Molecular Biology, University of Duisburg-Essen, Hufelandstrasse 55, 45122 Essen, Germany; 2 Department of Medical Microbiology, University of Duisburg-Essen, Virchowstrasse 179, 45147 Essen, Germany; 3 Department of Surgery, University of Cincinnati, Cincinnati, OH 45267 USA; 4 Department of Pharmacology and Toxicology, Medical College of Virginia, Virginia Commonwealth University, Richmond, VA 23298 USA

**Keywords:** Acid sphingomyelinase/ceramide system, Lung edema, Superoxide, *Staphylococcus aureus*

## Abstract

**Abstract:**

Pulmonary edema associated with increased vascular permeability is a severe complication of *Staphylococcus aureus*–induced sepsis and an important cause of human pathology and death. We investigated the role of the mammalian acid sphingomyelinase (Asm)/ceramide system in the development of lung edema caused by *S. aureus*. Our findings demonstrate that genetic deficiency or pharmacologic inhibition of Asm reduced lung edema in mice infected with *S. aureus*. The Asm/ceramide system triggered the formation of superoxide, resulting in degradation of tight junction proteins followed by lung edema. Treatment of infected mice with amitriptyline, a potent inhibitor of Asm, protected mice from lung edema caused by *S. aureus*, but did not reduce systemic bacterial numbers. In turn, treatment with antibiotics reduced bacterial numbers but did not protect mice from lung edema. In contrast, only the combination of antibiotics and amitriptyline inhibited both pulmonary edema and bacteremia protecting mice from lethal sepsis and lung dysfunction suggesting the combination of both drugs as novel treatment option for sepsis.

**Key messages:**

- Antibiotics are often insufficient to cure *S. aureus*–induced sepsis.
- *S. aureus* induces lung edema via the Asm/ceramide system.
- Genetic deficiency of Asm inhibits lung dysfunction upon infection with *S. aureus*.
- Pharmacologic inhibition of Asm reduces lung edema induced by *S. aureus*.
- Antibiotics plus amitriptyline protect mice from lung edema and lethal *S. aureus* sepsis.

**Electronic supplementary material:**

The online version of this article (doi:10.1007/s00109-014-1246-y) contains supplementary material, which is available to authorized users.

## Introduction

Although *Staphylococcus aureus* (*S. aureus*) is a commensal of the skin, it is also one of the most serious pathogens and often causes not only local purulent wound infections of the skin and soft tissues but also severe diseases such as pneumonia, endocarditis, osteomyelitis, sepsis, and toxic shock syndrome [1, 2]. In particular, the methicillin-resistant *S. aureus* (MRSA) strains are increasing in importance, particularly in hospitals [3, 4]. In the United States, *S. aureus* is responsible for approximately 500,000 infections and 20,000 deaths each year [5]. A dreaded complication of *S. aureus*–induced sepsis is lung edema, which is often fatal. Damage to the endothelium and disruption of tight junctions (TJs) are central to the pathophysiology of lung edema and sepsis after systemic infection with *S. aureus* [6, 7].

It has been previously shown that the interaction of *S. aureus* with endothelial cells depends on the activation of mammalian acid sphingomyelinase (Asm) [8]. The Asm is an ubiquitous enzyme, which is expressed in all cells including myeloid cells [9]. Asm hydrolyzes sphingomyelin, which is the most prominent sphingolipid in the membrane, to ceramide [10]. Ceramide has been shown to spontaneously form distinct domains in the plasma membrane leading to the formation of ceramide-rich platforms. Those platforms serve to trap and cluster receptor molecules, thereby permitting and amplifying signal transduction [11, 12]. This process may lead to subsequent apoptosis of the endothelial cells upon infection with *S. aureus*. However, several other pathogens besides *S. aureus*, such as *Neisseria gonorrhoeae*, *Listeria monocytogenes*, *Pseudomonas aeruginosa*, *Salmonella typhimurium*, *Escherichia coli*, and *Mycobacterium avium*, also exploit the Asm/ceramide system to infect mammalian cells [13–18]. The Asm/ceramide system has been shown to be involved in internalization of these pathogens, the induction of host cell apoptosis, the activation of intracellular signaling pathways, and the control of the release of cytokines.

The Asm/ceramide system is also crucially involved in killing bacteria, predominantly by facilitating the activation of NADPH oxidases. Ceramide domains cluster subunits of NADPH oxidases, thereby releasing superoxide after the infection of macrophages [19]. Although superoxide is necessary for killing bacteria, the results of previous studies also indicate that superoxide molecules contribute to *S. aureus*–induced dysfunction of the lung endothelial cell barrier [20]. The family of NADPH oxidases appears to be the predominant contributor of endothelial superoxide, which is activated by several stimuli and involved in diverse pathways, including endothelial cell activation and inflammation [21].

Here, we investigated the role of Asm in *S. aureus*–induced lung edema. Our findings provide evidence that the Asm/ceramide system plays a central role in *S. aureus*–induced pulmonary edema via induction of superoxide and thereby destruction of TJ proteins. Genetic or pharmacologic inhibition of Asm or production of superoxide reduced lung injury after *S. aureus* infection. Pharmacologic inhibition of Asm with amitriptyline, a functional inhibitor of the enzyme, inhibited lung edema but did not reduce bacterial numbers in mice infected with *S. aureus*. Antibiotics reduced bacterial numbers but did not protect mice from lung edema, a finding consistent with the clinical observation that patients with *S. aureus* sepsis experience severe lung edema despite treatment with antibiotics. Treating infected mice with a combination of antibiotics and amitriptyline reduced both pulmonary edema and bacteremia, thus protecting mice from lethal sepsis and lung dysfunction.

## Materials and methods

### Mice and cells

**Fig. 1:**
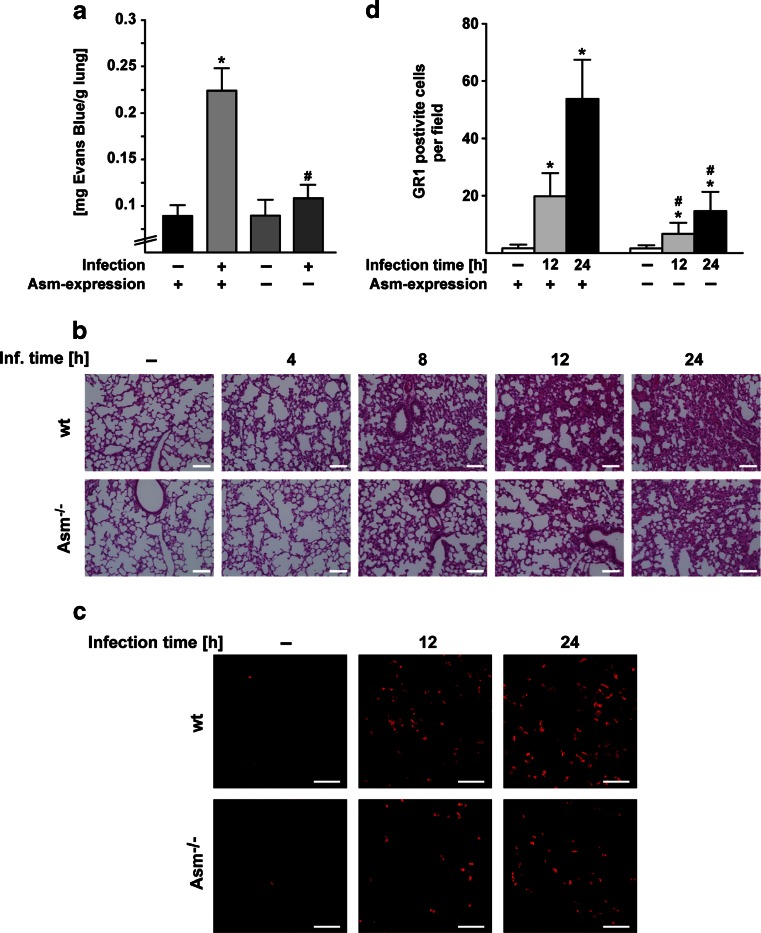
Acid sphingomyelinase deficiency protects against *S. aureus*–induced lung edema. **a** Wild-type (wt) and acid sphingomyelinase (Asm)-deficient mice were infected with *S. aureus* for 12 h. Evans Blue dye was injected 30 min before sacrificing the mice and removal of the lungs. The amount of dye leaking into the lung tissue was quantified. Shown are the mean ± SD of the concentration of Evans Blue dye in the lungs from each five wt and Asm-deficient mice. *Significant differences between uninfected mice and infected mice; #significant differences between infected wt mice and Asm-deficient mice (all *P* < 0.05; ANOVA). **b** Wt and Asm-deficient (Asm^−/−^) mice were infected with *S. aureus* for the indicated time periods. They were sacrificed, and lung sections were stained with H&E and analyzed by light microscopy for the detection of lung edema. *Scale bar* is 100 μm. Representative images from three independent experiments are shown. **c**, **d** For determination of pulmonary myeloid cell influx, wt and Asm-deficient mice were left uninfected or were infected with *S. aureus* for 12 or 24 h. Lung sections were stained with Cy3-labeled anti-GR1 antibodies and analyzed by fluorescence microscopy. *Scale bar* is 50 μm. Shown are representative images from three independent experiments. Cells staining positive for GR1, a myeloid cell marker, were quantified by analysis of 50 fields per group. Shown is the number (mean ± SD) of GR1-positive cells using a 630-fold magnification. *Significant differences between uninfected mice and infected mice; #significant differences between infected wt mice and Asm-deficient mice (all *P* < 0.05; *t* test).

Acid-sphingomyelinase (Asm)-deficient mice (sphingomyelin phosphodiesterase 1 knockout; *Smpd1* ^−/−^) and syngenic wild-type (wt) littermates on a C57BL/6J genetic background were kindly provided by Dr. R. Kolesnick (Memorial Sloan-Kettering Cancer Center, New York, NY, USA). Mice were maintained in the animal facility of the University Duisburg-Essen under pathogen-free conditions according to the criteria of the Federation of Laboratory Animal Science. The genotype was verified by PCR analysis before experimentation. For all experiments, we used mice only aged 6 to 8 weeks, which do not show an accumulation of myeloid cells, mostly macrophages, in the lung (Fig. 1c, non-infected sample). Further, the concentrations of TNF-α and IL-1 did not differ in lungs of 6-week-old wt and Asm-deficient mice (not shown). Infection experiments were approved by the Landesamt für Natur, Umwelt und Verbraucherschutz (LANUV), animal grant G1376/13 and local authorities.

For pretreatment with inhibitors before infection, wt mice were injected intraperitoneally with 10 mg/kg amitriptyline (Sigma-Aldrich, Deisenhofen, Germany), 100 mg/kg Tiron (Fluka Chemie GmbH, Buchs, Germany), or 100 mg/kg NAC (Sigma) twice daily for 2.5 days. The last dose was given 1 h before infection. For treatment with amitriptyline post infection, wt mice were injected i.p. 1 or 2 h after infection with 16 mg/kg amitriptyline. Antibiotics were also injected i.p. 1 h after infection with 100 mg/kg methicillin (Sigma) or 100 mg/kg vancomycin (Sigma). The injection of methicillin or vancomycin was repeated 9 h after infection. All mice, either pretreated with inhibitors or post-treated with amitriptyline and/or antibiotics, were sacrificed after 12 or 24 h. For survival experiments, we administered 16 mg/kg amitriptyline 1 h after infection and reduced to 10 mg/kg amitriptyline in the following injection after 12 h and then twice daily until 144 h post-infection. The antibiotics vancomycin and methicillin were administered twice daily (100 mg/kg), starting 12 and 24 h after infection until 144 h post-infection.

The in vitro experiments were performed with murine EOMA endothelial cells (ATCC® CRL-2586™), which were maintained in Dulbecco’s modified Eagle’s medium (DMEM) (Gibco/Invitrogen, Karlsruhe, Germany) supplemented with 10 % fetal calf serum (PAA, Pasching, Austria, A15-101), 10 mM HEPES (Roth GmbH, Karlsruhe, Germany), pH 7.4, 2 mM l-glutamine, 1 mM sodium pyruvate, 100 μM non-essential amino acids, 100 U/ml penicillin, and 100 μg/ml streptomycin (all from Thermo Fisher Scientific, Waltham, USA) at 37 °C in a 10 % CO_2_ atmosphere.

### Infection experiments

All in vivo and in vitro infections were performed with a clinical *S. aureus* strain isolated from a patient with sepsis. Further characterization of the strain showed that it produces alpha-toxin and enterotoxin D but not the Panton-Valentine leukocidin or toxic shock syndrome toxins. To exclude strain-specific results, we repeated the principal experiments with the well-characterized *S. aureus* sepsis strain Newman (ATCC® 25904) [43].

Bacteria were grown overnight on trypticase soy agar plates supplemented with 5 % sheep blood (BD Biosciences, Heidelberg, Germany). The plates were incubated for 16 h at 37 °C. Bacteria were then resuspended in prewarmed trypticase soy broth (TSB; BD) at an optical density (550 nm) of 0.2/ml, shaken at 125 rpm for 70 min at 37 °C, and collected during the early logarithmic growth phase by centrifugation at 3000 rpm for 10 min. The bacterial pellet was washed twice in DMEM supplemented with 10 mM HEPES (Roth GmbH, Karlsruhe, Germany) or phosphate buffered saline (PBS, 137 mM NaCl, 2.7 mM KCl, 7 mM CaCl_2_, 0.8 mM MgSO_4_, 1.4 mM KH_2_PO_4_, and 6.5 mM Na_2_HPO_4_) and then resuspended at 9 × 10^8^ colony-forming units (CFU) per milliliter of DMEM supplemented with 10 mM HEPES for infection of EOMA cells or at 5 × 10^6^ CFU per 100 μl in PBS for infection of mice.

EOMA cells were infected in DMEM/10 mM HEPES. We added 20 μM amitriptyline, 10 mM Tiron, or 10 mM NAC 20 min before infection and inoculated sub-confluent cell layers at a bacteria-to-host cell ratio (multiplicity of infection, MOI) of 10:1 or 200:1. Synchronous infection conditions and enhanced interactions between bacteria and host cells were achieved by a 2-min centrifugation (1000 rpm) of the bacteria onto the cells. The end of the centrifugation was defined as the starting point of infection. The infection was terminated by fixation or lysis, as described below.

To infect the mice, we intravenously injected 5 × 10^6^ CFU *S. aureus*. After the indicated time, the animals were sacrificed by cervical dislocation, and the lung, spleen, or liver was removed for further processing. To determine the amount of Evans Blue leakage into lung tissue, we injected mice intravenously with 4 % Evans Blue dye (20 mg/kg, Sigma) 30 min before sacrificing the mice (infection time 12 h); lungs were flushed with saline via the right heart, removed, dried, extracted in formamide (Sigma), centrifuged, and supernatants were measured at 620 and 740 nm with a fluorescence microplate reader (BMG Labtech, Offenburg, Germany). To determine neutrophil immigration capacity, we infected mice with 5 × 10^6^ CFU *S. aureus* i.p. for 2 h, sacrificed them, and injected ice-cold PBS into the peritoneal cavity. Peritoneum was then gently massaged and PBS was aspirated from the peritoneal cavity. The cell number was counted and 1 ml cell suspension was preincubated with anti-CD16/CD32 mAb (Biolegend, clone 93) to block FcRII/III receptors and stained for 15 min at 4 °C with FITC-anti-mouse Ly-6G (Gr1) (eBioscience, clone RB6-8C5), PE-Cy7-anti-mouse CD11b (eBioscience, clone M1/70), and APC-anti-mouse F4/80 (eBioscience, clone BM8). Cells were analyzed using FACS Calibur™ (BD Bioscience) and absolute numbers were calculated.

### Assay for acid sphingomyelinase activity

The activity of Asm was measured as the conversion of radioactive [^14^C]-sphingomyelin to ceramide and [^14^C]-phosphorylcholine. Then 8 × 10^5^ EOMA cells were infected with *S. aureus* for the indicated time periods, washed, and lysed in 300 μl ice-cold Asm lysis buffer [1 % NP-40, 250 mM sodium acetate (pH 5.0)] per sample, aliquots diluted to 0.1 % NP-40 and 250 mM sodium acetate (pH 5.0), and incubated with [^14^C]-sphingomyelin (Perkin Elmer, specific activity = 2 GBq/mmol) for 30 min at 37 °C. [^14^C]-sphingomyelin was dried prior to use and solubilized into micelles in 0.1 % NP-40, 250 mM sodium acetate (pH 5.0) using a bath sonicator for 10 min. Samples were normalized for protein using Bradford assays.

Cell lysates were incubated for 60 min with 0.05 μCi [^14^C]-labeled sphingomyelin per sample at 37 °C with a thermomixer (Eppendorf AG, Hamburg, Germany). Lipids were then extracted by the addition of 1 ml CHCl_3_/CH_3_OH (2:1, v/v) per sample, followed by vortexing for 15 s and centrifugation at 14,000 rpm for 5 min. An aliquot of the aqueous phase was applied for liquid scintillation counting. Hydrolysis of [^14^C]-sphingomyelin by Asm results in the release of [^14^C]-choline chloride into the aqueous phase, whereas ceramide and unreacted [^14^C]-sphingomyelin remain in the organic phase. Therefore, the release of [^14^C]-choline chloride serves to determine the activity of Asm.

### Ceramide measurements

Ceramide was measured using a DAG kinase assay, as previously described [15, 17]. Briefly, cells were infected for the indicated time and then extracted in CHCl_3_/CH_3_OH/1 N HCl (100:100:1, v/v/v). The organic phase was collected and dried lipids were solubilized in 20 μl of 7.5 % *N*-octyl glucopyranoside, 5 mM cardiolipin, 1 mM DETAPAC and sonicated in a bath sonicator for 10 min. We then added 50 μl DAG kinase reaction buffer [100 mM imidazole/HCl (pH 6.6), 100 mM NaCl, 25 mM MgCl_2_, 2 mM EDTA, 2.8 mM DTT, 5 μM ATP, 10 μCi [^32^P]-γATP] and 0.01 U DAG kinase (BML-SE100; Enzo Life Sciences, USA) in 10 μl of 1 mM DETAPAC (pH 6.6) and 0.01 M imidazole. The kinase reaction was performed for 30 min at room temperature.

The samples were then re-extracted in 1 ml CHCl_3_/CH_3_OH/1 N HCl (100:100:1, v/v/v), 170 μl buffered salt solution [135 mM NaCl, 1.5 mM CaCl_2_, 0.5 mM MgCl_2_, 5.6 mM glucose, 10 mM HEPES (pH 7.2)], and 30 μl of a 100 mM EDTA solution per sample, followed by vortexing. The lower organic phase was concentrated, and dried lipids were dissolved in 20 μl CHCl_3_/CH_3_OH (1:1, v/v) per sample and separated on a Silica G60 thin layer chromatography (TLC) plate (Merck, Darmstadt, Germany) with a solvent system consisting of CHCl_3_/CH_3_COCH_3_/CH_3_OH/CH_3_COOH/H_2_O (10:4:3:2:1, v/v/v/v/v). The plate was analyzed using a Fuji phosphoimager. Ceramide spots were identified by co-migration with a C_16_/C_24_-ceramide standard. Comparison with a standard curve for C_16_/C_24_-ceramide permitted the determination of ceramide amounts.

To determine local accumulation of ceramide after infection, EOMA cells were infected with *S. aureus* (MOI 10:1) for 40 min, washed twice in PBS, and fixed with 1 % PFA in buffered PBS (pH 7.3) for 15 min. The cells were then washed in PBS, permeabilized for 10 min with 0.1 % Triton X-100 in PBS, washed in PBS, blocked with an irrelevant affinity-purified donkey antibody (Jackson; 1:100 in PBS + 0.025 % Tween 20) for 60 min at room temperature, washed three times with PBS + 0.025 % Tween 20, and incubated with a rabbit anti-*S. aureus* antibody (Abcam 20920; 1:500 in PBS + 0.025 % Tween 20 + 5 % FCS) for 60 min at room temperature. The cells were then washed three times with PBS + 0.025 % Tween 20 and incubated with Alexa-Fluor 647-coupled anti-rabbit Ig (Jackson; 1:500 in PBS + 0.025 % Tween 20 + 5 % FCS) overnight at 4 °C. The samples were washed again three times, blocked again as above, washed three times followed by a 60-min staining at room temperature with mouse monoclonal anti-ceramide IgM (Glycobiotech MAB 0011; 1:100 in PBS + 0.025 % Tween 20 + 5 % FCS). The samples were washed three times, stained with Cy3-labeled anti-mouse IgM (Jackson; 1:500 in PBS + 0.025 % Tween 20 + 5 % FCS) for 60 min at room temperature, washed three times, and embedded in Mowiol (Kuraray Specialities Europe GmbH, Frankfurt, Germany). The samples were analyzed by confocal microscopy using a Leica TCS SL microscope (Leica, Mannheim, Germany).

### Measurement of production of superoxide

Superoxide production was measured by electron spin resonance (ESR), as described previously [31]. Then 2 × 10^5^ endothelial cells were infected with *S. aureus* for the indicated time, the medium removed, the cells scraped into 20 mM HEPES (pH 7.5), 1 mM EDTA, and 255 mM sucrose, and shock frozen in liquid nitrogen. Proteins were isolated and resuspended with modified Krebs-HEPES buffer containing deferoximine (100 μM, Sigma) and diethyldithiocarbamate (5 μM, Sigma). A spin trap, 1-hydroxy-3-methoxycarbonyl-2,2,5,5-tetramethylpyrrolidine (CMH, Noxygen, Elzach, Germany) (1 mM final concentration), was then added to the mixture in the presence or absence of manganese-dependent superoxide dismutase (SOD, 200 U/ml; Sigma, St. Louis, MO). The mixture was loaded into glass capillaries and immediately kinetically analyzed for O_2_^-.^ production for 10 min. The SOD-inhibited fraction of the signal was used to calibrate the system. The ESR settings were as follows: biofield, 3350; field sweep, 60 G; microwave frequency, 9.78 GHz; microwave power, 20 mW; modulation amplitude, 3 G; points of resolution, 4096; receiver gain, 100; and kinetic time, 10 min. The ESR signal strength was recorded in arbitrary units and the final results were expressed as the fold changes compared to the control.

### Immunocytochemistry

Cells were grown on coverslips, infected or left uninfected, fixed in 2 % paraformaldehyde (PFA, Sigma) buffered in PBS (pH 7.2–7.4) for 10 min, and washed in PBS. For intracellular staining of ZO1, ZO2, occludin, and E-cadherin, cells were permeabilized with 0.1 % Triton X-100 (Sigma) in PBS (pH 7.4) for 5 min at room temperature, washed with PBS, and blocked for 1 h in PBS supplemented with 5 % fetal calf serum (FCS, Gibco). Samples were washed and incubated overnight at 4 °C with antibodies against ZO1 (Invitrogen 40-2300, rabbit IgG), ZO2 (Santa Cruz Biotechnology Inc. sc-11448, rabbit IgG), occludin (71-1500, rabbit IgG, Invitrogen), or E-cadherin (Santa Cruz Biotechnology Inc. sc-7870, rabbit IgG). Cells were washed three times in PBS with 0.05 % Tween 20 (Sigma), incubated for 1 h with Cy3-labeled donkey anti-rabbit antibodies, and washed again in PBS with 0.05 % Tween 20. After a final wash with PBS, cells were mounted on glass coverslips with Mowiol. Cells were examined with a Leica TCS SP5 confocal microscope (Leica, Mannheim, Germany) using Leica software, version 2.61.

### Histopathologic assessment

Mice were sacrificed, the lungs were removed, fixed in 4 % PFA for 38 h, serially dehydrated, and embedded in paraffin for sectioning at 6 μm. For stainings, sections were dewaxed and rehydrated. H&E staining was performed by incubating lung sections in hematoxylin for 2 min and in eosin for 1 min. For fluorescence staining, lung sections were incubated in pepsin (Invitrogen) for 20 min at 37 °C, washed, and treated for 10 min in PBS supplemented with 5 % FCS and 0.5 % Tween 20. Lung sections were exposed overnight at 4 °C to anti-ZO1 IgG, anti-ZO2 IgG, anti-E-cadherin IgG, anti-occludin IgG, or anti-Ly-6G and Ly-6C (Gr-1, rat IgG; BD) antibodies (all antibodies as above). The sections were washed in PBS with 0.05 % Tween 20 and then incubated for an additional 45 min with Cy3-labeled secondary antibodies (Jackson ImmunoResearch, West Grove, PA, USA). For co-stainings of TJs and endothelial cells, samples were incubated together with antibodies against ZO1, ZO2, occludin, and E-cadherin additionally with FITC-Lectin (Fluorescein Griffona Simplicifolia Lectin I-Isolectin B_4_, Vector, FL-1201, 1:50) and then proceeded as above. The sections were washed once in PBS with 0.05 % Tween 20 and once in PBS and were mounted in Mowiol. Lung samples were analyzed by light transmission or confocal microscopy.

### Quantification of *S. aureus* colony-forming units in liver, spleen, and lung

To quantify *S. aureus* colony-forming units (CFU), we removed the liver, spleen, and lung from each mouse after the indicated time of infection and homogenized them in a loose Dounce homogenizer (Braun, Germany). The homogenates were lysed for 10 min in 5 mg/ml saponin (Serva Electrophoresis GmbH, Heidelberg, Germany) at 37 °C for the release of intracellular bacteria. Samples were centrifuged for 10 min at 3200 rpm, resuspended in PBS, and plated on lysogeny broth (LB) plates. Bacterial CFU were counted after the plates had been incubated overnight at 37 °C.

### Statistical analysis

All data are displayed as mean ± SD. All data were tested for normal distribution using the David–Pearson–Stephens test. Statistical analysis was performed with Student’s *t* test for single comparisons and ANOVA for multiple comparisons. Groups in the survival tests were compared by log-rank test. The sample size planning for the continuous in *S. aureus* in vivo infections experiments was based on two-sided Wilcoxon–Mann–Whitney tests using the free software G*Power version 3.1.7 of the University of Dusseldorf, Germany. Statistical significance was set at the level of *P* <0.05.

## Results

### Acid sphingomyelinase deficiency reduces *S. aureus*–induced lung edema

To determine whether Asm plays a role in the in vivo development of lung edema caused by *S. aureus* infection, we systemically infected C57BL/6 wild-type (wt) and Asm-deficient mice with *S. aureus* for various time periods. To quantify lung edema, we injected the dye Evans Blue, which leaks into the lung only if the endothelial cell integrity is disrupted and thereby serves to quantify lung edema in vivo. The studies revealed massive leakage of Evans Blue dye into the lungs of wt mice but almost no leakage into the lungs of Asm-deficient mice after systemic infection with a clinical *S. aureus* strain (Fig. 1a) or *S. aureus* Newman (supporting Fig. 1A). Moreover, hematoxylin and eosin (H&E) staining of the lungs demonstrated that *S. aureus* infection induces lung edema in wt mice as indicated by thick hyaline walls in large venules and massive cellular infiltrates, findings that were absent or much less pronounced in Asm-deficient mice (Fig. 1b and supporting Fig. 1B, C). The absence of lung edema in Asm-deficient mice correlated with a reduced number of myeloid cells in the lungs of Asm-deficient mice, while high numbers of GR1-positive, myeloid cells were observed in the lungs of *S. aureus*–infected wt mice (Fig. 1c, d). Controls revealed that Asm deficiency did not affect the ability of myeloid cells to migrate (supporting Fig. 1D), but reduced the production of superoxide in myeloid cells (supporting Fig. 1E).

Taken together, these findings indicate that Asm plays a key role in the development of pulmonary edema induced by systemic infection with *S. aureus*. Asm deficiency inhibits the development of lung injury during infection.

### Infection of endothelial cells with *S. aureus* activates Asm and leads to the production of superoxide in a positive feedback loop

**Fig. 2:**
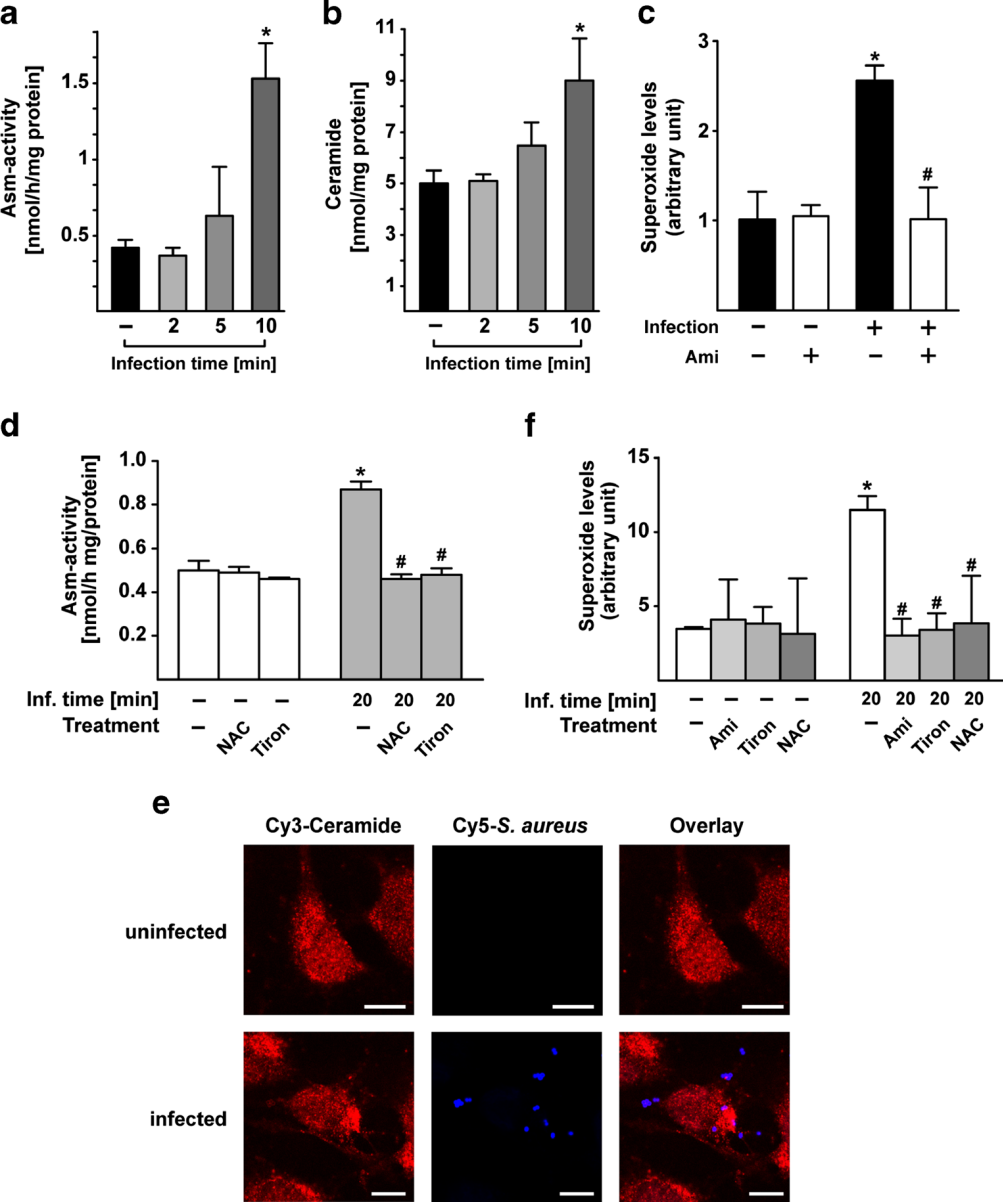
Asm is necessary for *S. aureus*–induced production of superoxide. **a**, **b** Asm activity (**a**) and ceramide concentrations (**b**) were measured in EOMA cells after infection with *S. aureus* for 0, 2, 5, or 10 min at an MOI of 200:1. Results show the mean ± SD of three independent experiments. *Significant differences compared to uninfected control mice (*P* < 0.05, *t* test). **c** EOMA cells were infected with *S. aureus* for 7.5 min at an MOI of 200:1. The production of superoxide was quantified by electron spin resonance. Relative O_2_^-.^ levels were used to indicate superoxide accumulation. Shown are means ± SD from four independent experiments. *Significant differences between uninfected samples and infected samples; #significant differences between untreated samples and amitriptyline (Ami)-treated samples (all *P* < 0.05, ANOVA). **d–f** Infection with a lower MOI (10:1) of *S. aureus* also resulted in Asm activation (**d**), ceramide accumulation at the site of *S. aureus* infection (**e**), and superoxide release (**f**). Pre-incubation of EOMA cells with the antioxidants Tiron and *N*-acetylcysteine (NAC) (each 10 mM) reduced Asm activation (**d**). Pre-incubation of EOMA cells with the functional Asm inhibitor amitriptyline or with antioxidants also inhibited superoxide release (**f**). Shown are means of arbitrary units ± SD or representative figures from four independent experiments. *Scale bar* is 10 μm. *Significant differences between uninfected samples and infected samples; #significant differences between untreated and treated samples (all *P* < 0.05, ANOVA). Figures 3 and 4. *S. aureus* induces disruption of TJs in endothelial cells via Asm-mediated superoxide production

To further define the role of the Asm in lung edema induced by systemic infections with *S. aureus*, we infected murine endothelial (EOMA) cells with *S. aureus* and measured Asm activity. *S. aureus* infection induced a marked activation of the Asm (Fig. 2a and supporting Fig. 2A) and formation of ceramide (Fig. 2b and supporting Fig. 2B), the product of Asm activity.

Superoxide has long been known to play a crucial role in host-pathogen interactions [20, 22, 23]. To determine whether *S. aureus* infection also induces the release of superoxide and whether this superoxide release depends on Asm, we infected EOMA cells and analyzed the production of oxygen radicals. The results showed that *S. aureus* infection induces a rapid production of superoxide in EOMA cells (Fig. 2c and supporting Fig. 2C). Infection with a lower multiplicity of infection (MOI) of *S. aureus* revealed a similar but slightly delayed time course of Asm activation (Fig. 2d), intracellular ceramide accumulation at the site of *S. aureus* internalization (Fig. 2e), and superoxide release (Fig. 2f). Pre-incubation of EOMA cells with the antioxidants Tiron and *N*-acetylcysteine (NAC) reduced Asm activation by *S. aureus* (Fig. 2d). Pre-incubation of EOMA cells with the functional Asm inhibitor amitriptyline (Ami) [17, 24, 26] or with Tiron and NAC also inhibited superoxide release (Fig. 2c, f and supporting Fig. 2C) suggesting a positive feedback loop of *S. aureus*–induced Asm activation and superoxide release.

### *S. aureus* induces degradation of TJs via the Asm/ceramide system

**Fig. 3:**
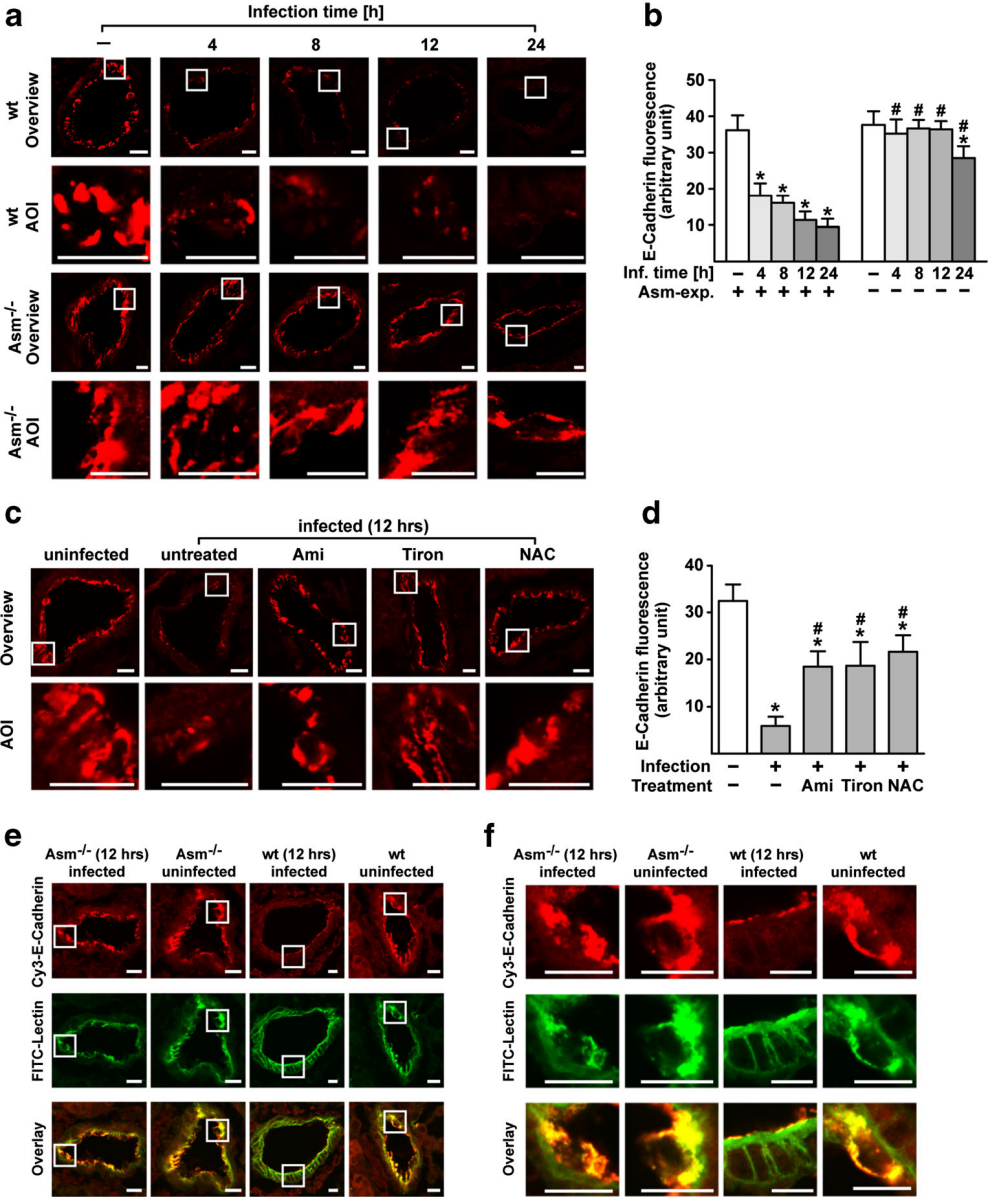
Wild-type (wt) and Asm-deficient mice (Asm^−/−^) were left uninfected or were infected with *S. aureus* for the indicated time points. Wt mice were pretreated before infection with amitriptyline (Ami) (10 mg/kg) or the antioxidants Tiron (100 mg/kg) and *N*-acetylcysteine (NAC) (100 mg/kg), or left untreated and/or uninfected. Lung sections were stained with Cy3-coupled antibodies against E-cadherin (**a**, **c**) or co-stained for cadherin and the endothelial cells marker FITC-lectin (**e**, **f**) and analyzed by confocal microscopy for determination of the degradation of these TJ proteins. Representative images from three independent experiments are shown [original image and an area of interest (AOI)]. *Scale bar* is 10 μm. To score TJ protein fluorescence, we focused on one large vessel, chose the strongest fluorescence, and analyzed signal intensity in ten pictures per lung. The investigators were blinded to the identity of the samples (**b**, **d**). *Significant differences between uninfected and infected samples; #significant differences between wt and Asm-deficient or untreated and treated samples (all *P* < 0.05, ANOVA).

To gain insight into the mechanism by which Asm and ceramide mediate endothelial dysfunction and lung edema after *S. aureus* infection, we determined whether systemic infection with *S. aureus* induces the breakdown of TJs in pulmonary endothelial cells in vivo and, if so, whether this process depends on the Asm/ceramide system. To this end, we systemically infected wt and Asm-deficient mice with *S. aureus*. We then obtained lung sections and stained them with Cy3-labeled antibodies to ZO1, ZO2, occludin, or E-cadherin. Confocal microscopy showed that infection with *S. aureus* induces dramatic degradation of TJ proteins in a time-dependent manner in endothelial cells from blood vessels in wt lungs but not in endothelial cells from the lungs of Asm-deficient mice or wt mice pretreated with amitriptyline (Fig. 3a–d and supporting Fig. 3A–H). Co-stainings of lung sections with Cy3-coupled antibodies against TJs and FITC-isolectin B_4_, which is a marker for endothelial cells, confirmed that TJ proteins are only degraded in lung endothelial cells of wt mice upon infection with *S. aureus*, but not in Asm-deficient endothelial cells [25] (Fig. 3e, f and supporting Fig. 4A–F).

**Fig. 4:**
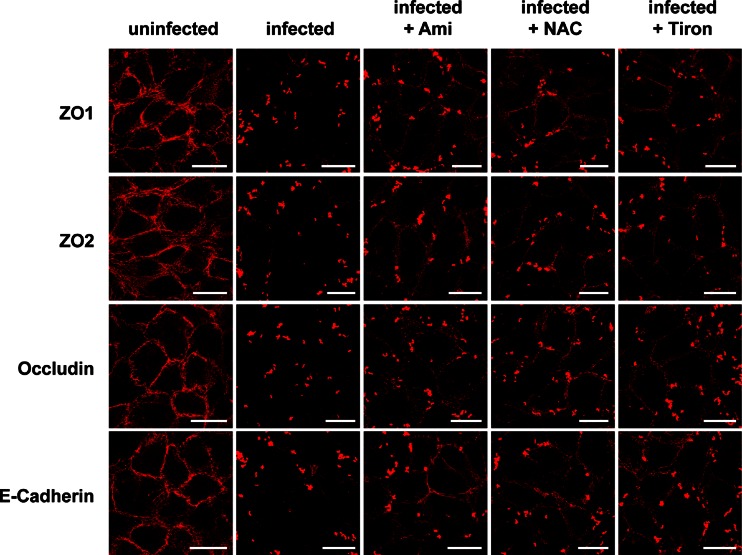
Endothelial cells were infected for 2 h with *S. aureus* (MOI 10:1) or left uninfected. As indicated, cells were pretreated for 20 min with amitriptyline (Ami) (20 μM), Tiron (10 mM), or NAC (10 mM) before infection with *S. aureus*. Immunofluorescence stainings were performed with antibodies against ZO1, ZO2, occludin, or E-cadherin for determination of the degradation of these TJ proteins. The presented pictures are representative of the results of at least three independent experiments. *Scale bar* is 25 μm

To determine whether TJs degradation pre-supposes the production of superoxide, we pretreated wt mice with intraperitoneal injections of both Tiron or NAC and then infected them with *S. aureus*. Confocal microscopy analysis demonstrated that the inhibition of superoxide also protects TJ proteins in endothelial cells from degradation after systemic *S. aureus* infection in vivo (Fig. 3c, d and supporting Fig. 3B, D, F, H). The in vivo findings were confirmed by in vitro studies: infecting EOMA cells with *S. aureus* induces a marked degradation of the TJ proteins ZO1, ZO2, occludin, and E-cadherin, changes that were reduced by pre-incubation with inhibition of the Asm by amitriptyline or superoxides by NAC or Tiron (Fig. 4 and supporting Fig. 5A, B).

Collectively, these findings suggest that Asm mediates the *S. aureus*–induced breakdown of TJ proteins by superoxide in vivo.

### Pharmacologic inhibition of Asm or superoxide reduces lung edema after systemic infection with *S. aureus*

**Fig. 5:**
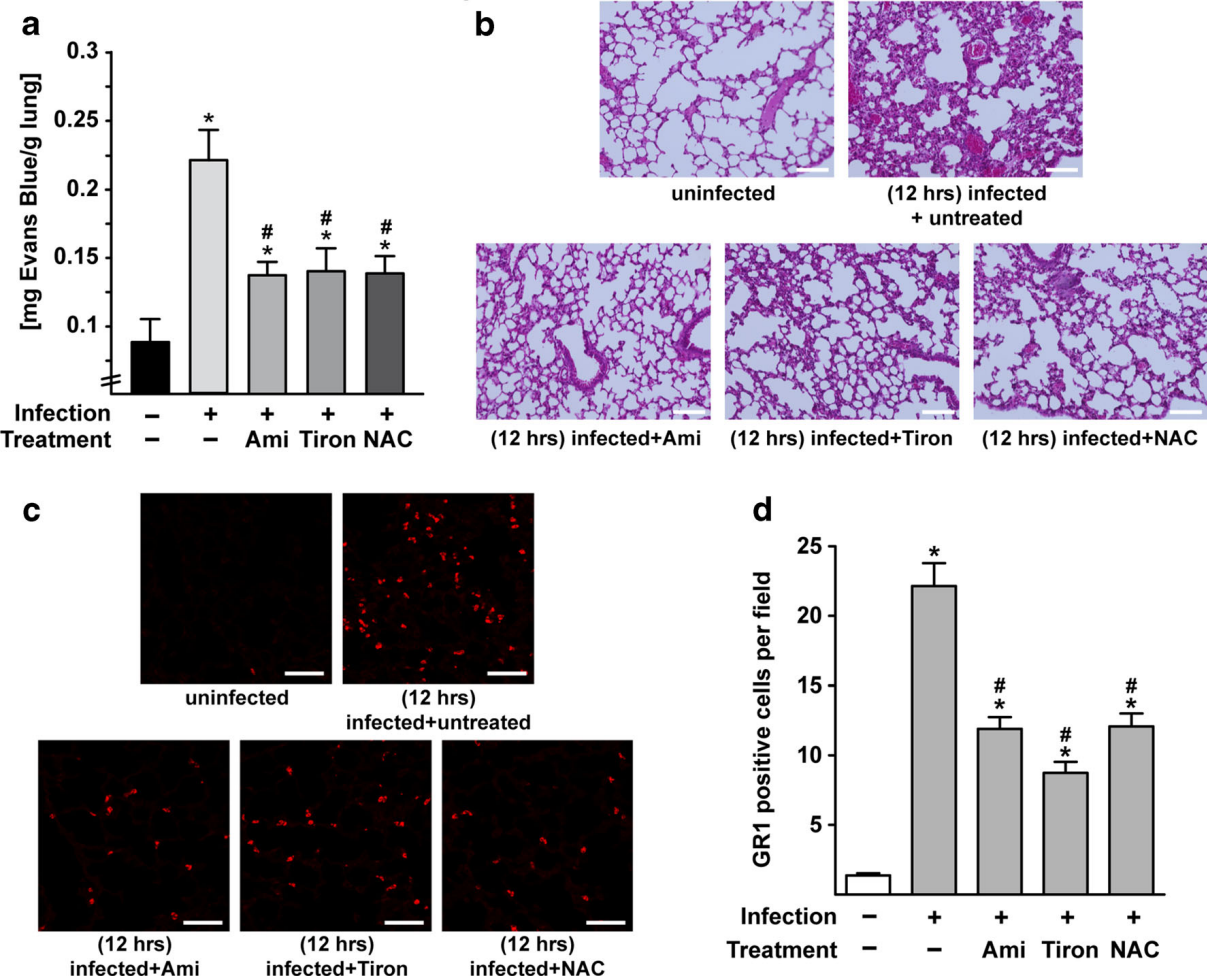
Pharmacologic inhibition of acid sphingomyelinase (Asm) or superoxide reduces pulmonary edema caused by systemic infection with *S. aureus.* Wild-type (wt) mice were pretreated by intraperitoneal injection of 10 mg/kg amitriptyline (Ami) or 100 mg/kg Tiron/*N*-acetylcysteine (NAC), respectively. Mice were infected with *S. aureus* for 12 h. Lung edema was determined by extravasation of Evans Blue (**a**) and by staining with H&E (*scale bar* is 100 μm) (**b**). Myeloid cell emigration was determined by staining of lung sections with Cy3-labeled anti-GR1 antibodies (*scale bar* is 50 μm) followed by fluorescence microscopy (**c**). Shown are representative images from three independent experiments. Myeloid cell influx was quantified by analysis of 50 fields per group (**d**). Displayed is the average of GR1-positive cells per field using a 630-fold magnification. *Significant differences between uninfected and infected samples; #significant differences between untreated and treated samples (all *P* < 0.05, ANOVA).

To test the significance of the pathway from the Asm via superoxide to the degradation of TJ proteins and the development of lung edema, we treated wt mice with intraperitoneal injections of amitriptyline or the antioxidants Tiron and NAC before systemic infection with *S. aureus* and then measured lung edema and myeloid cell influx after 12 h. *S. aureus* infection induced severe lung edema and influx of myeloid cells into the lung, events that were inhibited by pretreatment with amitriptyline, Tiron or NAC (Fig. 5a–d). These findings show that lung edema induced by *S. aureus* via the pathway through Asm and superoxide can be reduced by pretreatment with pharmacologic inhibitors of this pathway.

### Treatment of already septic mice with amitriptyline reduces the development of lung edema

**Fig. 6:**
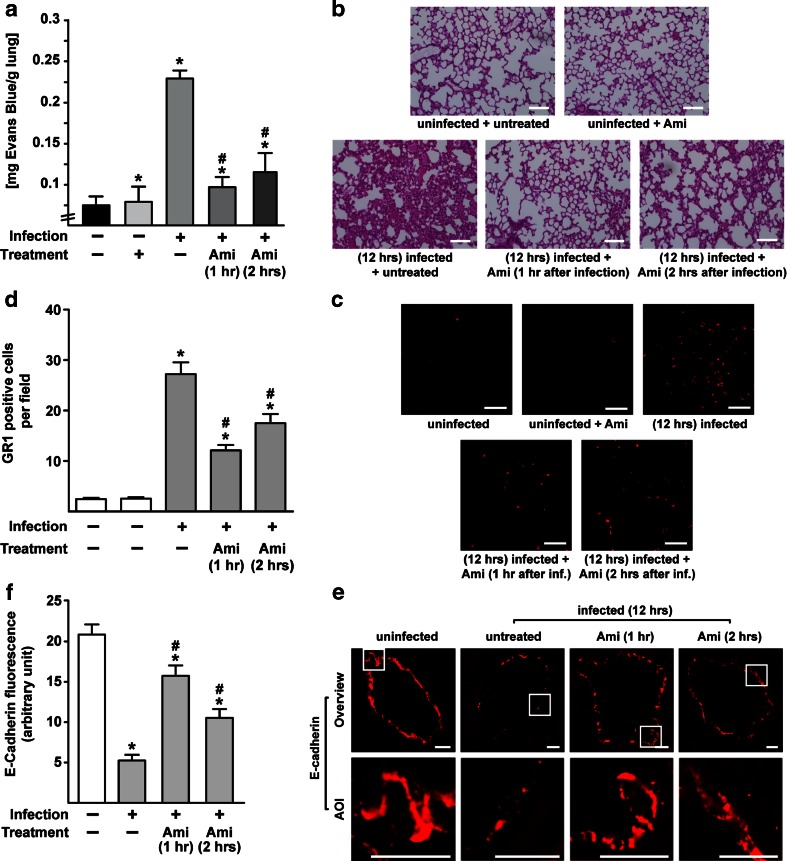
Treatment of already infected mice with amitriptyline protects against lung edema (**a–d)**. Wild-type (wt) mice were infected with *S. aureus*. Then 1 or 2 h later, they were i.p. injected with 16 mg/kg amitriptyline (Ami). The mice were sacrificed 12 h after infection. The lungs were removed and lung edema and myeloid cell trafficking were determined by extravasation of Evans Blue (**a**), or staining with H&E (*scale bar* is 100 μm) (**b**) or with Cy3-labeled anti-GR1 antibodies (*scale bar* is 50 μm) (**c**). The average number of GR1-positive cells per field using a 630-fold magnification was quantified by analysis of 50 fields per group (**d**). The breakdown of TJs was analyzed by staining lung sections with Cy3-labeled anti-E-cadherin antibodies (*scale bar* is 10 μm) (**e**). Panel (**a**) shows the mean ± SD from four mice. Images in (**b**), (**c**), and (**e**) are representative from three independent experiments. TJ fluorescence was scored via Photoshop (ten pictures per mouse) (**f**). Data in (**d**) and (**f**) are shown as mean ± SD, *n* = 3. *Significant differences between uninfected and infected samples; #significant differences between treated and untreated samples (all *P* < 0.05, ANOVA).

The finding that both pre-incubation with the Asm inhibitor amitriptyline and Asm deficiency protect mice from lung edema induced by *S. aureus* infection led us to question whether the administration of amitriptyline reduces the severity of pulmonary edema in mice that were already infected with *S. aureus*. If so, amitriptyline administration might be a clinically relevant therapeutic option for the treatment of *S. aureus–*induced pulmonary edema. To this end, we infected wt mice with *S. aureus* and treated them with amitriptyline (Ami) 1 or 2 h later. The mice were sacrificed 12 h after infection and Evans Blue extravasation into lung tissue was determined or lung sections were stained with H&E. Treatment with amitriptyline reduced the severity of pulmonary edema and the influx of myeloid cells even after the onset of systemic infection with *S. aureus* (Fig. 6a–d) and the breakdown of TJ proteins (Fig. 6e, f and supporting Fig. 6A–D).

Thus, treatment with amitriptyline reduces the severity of lung edema even when the drug is administered 1 or 2 h after systemic infection with *S. aureus*.

### The combination of amitriptyline and antibiotics inhibits sepsis

Clinically, treating *S. aureus* sepsis with antibiotics achieves only limited success, and severe lung edema often still develops even with appropriate antibiotic treatment [27–29]. Thus, with the aim of developing novel efficient therapeutic approaches to the treatment of *S. aureus*–induced sepsis and lung edema, we examined the effect of a combination of amitriptyline and antibiotics on bacterial killing and lung edema after systemic *S. aureus* infection. To this end, we infected wt mice with *S. aureus*. With the incidence of first clinical symptoms 1 h after infection, amitriptyline or methicillin or vancomycin or a combination of amitriptyline and either methicillin or vancomycin were injected. The injection of methicillin or vancomycin was repeated after 9 h. Control mice were left uninfected. The mice were sacrificed 24 h after infection, and bacterial numbers were determined in liver, spleen, and lung.

**Fig. 7:**
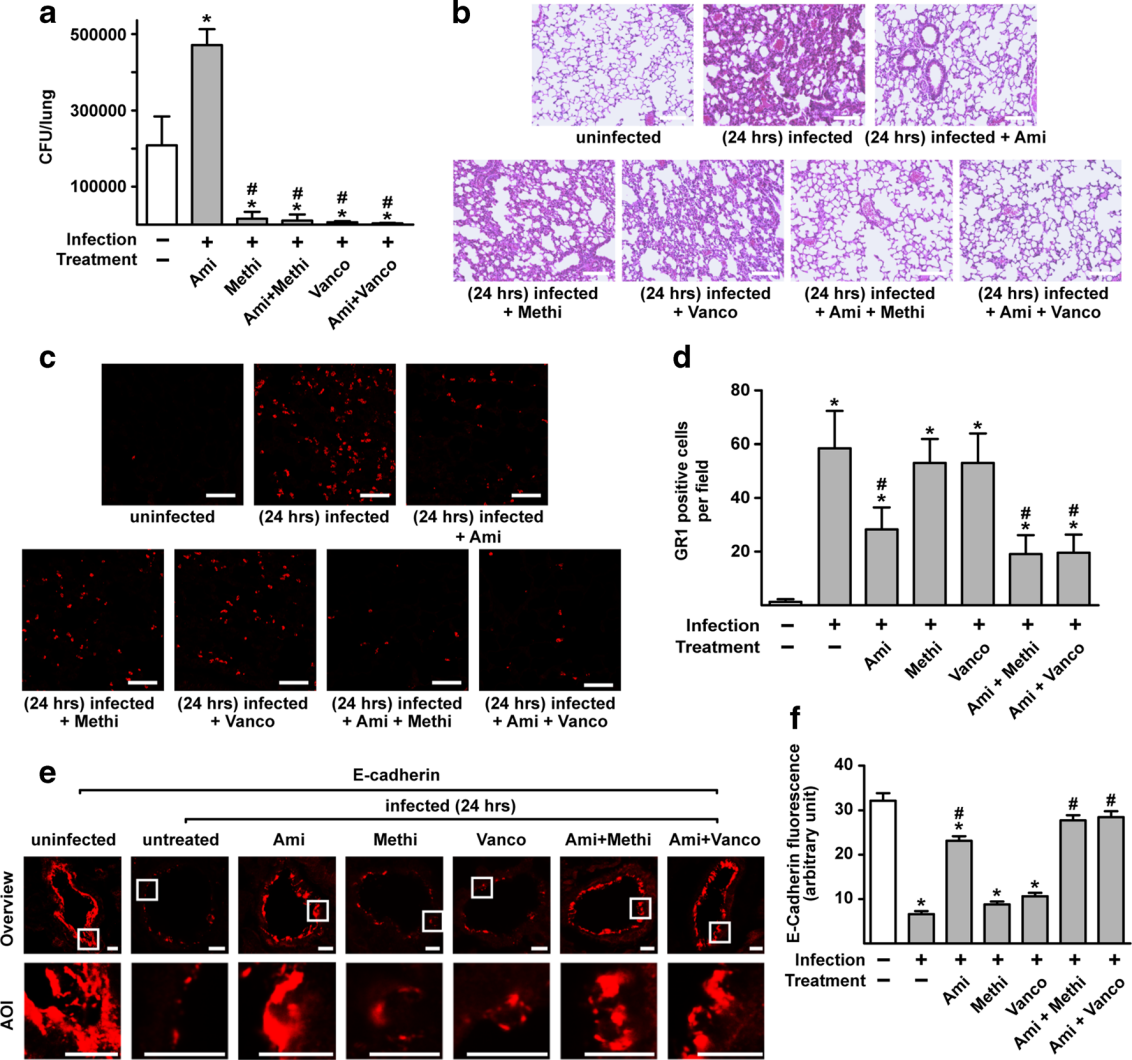
The combination of amitriptyline and antibiotics cures *S. aureus* sepsis and inhibits lung edema Wt mice were infected with *S. aureus*. They were then left untreated, treated with an i.p. injection of amitriptyline (*Ami*) (16 mg/kg) 1 h after infection, treated with either methicillin (*Methi*) or vancomycin (*Vanco*) (both 100 mg/kg) 1 and 9 h after infection, or treated with the combination of amitriptyline and methicillin or vancomycin. The mice were sacrificed 24 h after infection. **a** Lungs were removed, homogenized, and lysed in saponin for the quantification of intracellular and extracellular bacteria (colony-forming units, CFU) on lysogeny broth (LB) plates. Data shown are mean ± SD of three independent experiments from the lung; very similar data were obtained for bacterial counts in the liver and spleen (not shown). *Significant differences between uninfected and infected samples; #significant differences between treated and untreated samples (*P* < 0.05, ANOVA). **b** Lungs from four mice per group were obtained after infection and subsequent treatment with the indicated drugs. Microscopic analysis of lung sections stained either with H&E for lung edema (*scale bar* is 100 μm), with Cy3-labeled anti-GR1 antibody for myeloid cell trafficking (*scale bar* is 50 μm) (**c**), or for disruption of TJs with Cy3-labeled anti-E-cadherin antibodies (**e**) (*scale bar* is 10 μm) was performed. Shown are representative images from three independent experiments by light transmission or confocal microscopy (**b**, **c**, **e**). The average number of GR1-positive cells per field using a 630-fold magnification was analyzed of 50 fields per group (**d**). TJ fluorescence was scored via Photoshop (ten pictures per lung) (**f**). Data in (**d**) and (**f**) are shown as mean ± SD, *n* = 3. *Significant differences between uninfected and infected samples; #significant differences between treated and untreated samples (all *P* < 0.05, ANOVA).

While antibiotics alone or in combination with amitriptyline kill bacteria in liver, spleen, and lung, neither methicillin nor vancomycin alone reduced lung edema. In contrast, amitriptyline alone inhibited lung edema but did not reduce bacterial numbers. Only the combination of amitriptyline with antibiotics reduced bacteremia, lung edema, pulmonary influx of myeloid cells, and disruption of tight junctions (Fig. 7a–f; supporting Fig. 7A–F; supporting Fig. 8A–G). Control studies confirmed that the drugs were without effect on lung parameters in uninfected mice (supporting Fig. 9A–F).

These findings indicate that treating *S. aureus* infection with a combination of amitriptyline and antibiotics might be an effective clinical treatment for *S. aureus*–induced sepsis because such treatment reduced both bacterial burden and lung edema.

### The pharmacological treatment of lung edema and bacterial burden protects from lethality of *S. aureus* sepsis

**Fig. 8:**
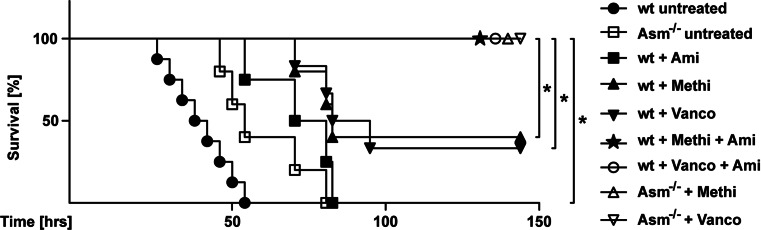
The pharmacological treatment of lung edema and bacterial burden protects from lethality of *S. aureus* sepsis. For mortality experiments, wild-type (wt) and Asm-deficient (Asm^−/−^) mice were infected intravenously with 5 × 10^6^ CFU *S. aureus*. Mice were then left untreated or were pharmacologically treated with amitriptyline (*Ami*) (1 h after infection, then twice daily), methicillin (*Methi*), or vancomycin (*Vanco*) (1 and 9 h after infection and then twice daily) or with a combination of amitriptyline and antibiotics. Survival was observed for up to 6 days. Data are shown in percent survival. Significance was determined by log-rank (Mantel–Cox) test.

To investigate the link between bacterial burden and sepsis-induced lethality, we performed mortality experiments with untreated and pharmacologically treated wt and Asm-deficient mice after infecting them intravenously with 5 × 10^6^ CFU *S. aureus*. Wt mice died between 26 and 52 h after infection. Treatment of wt mice with amitriptyline delayed the death of the mice and the mice died between 50 and 85 h after infection. A very similar time course was observed for Asm-deficient mice that died between 50 and 80 h of infection. Treatment of wt mice with methicillin or vancomycin (1 and 9 h after infection and then twice daily) alone only rescued 50 % mice (11-day observation period). In contrast, the treatment of wt mice with a combination of amitriptyline and antibiotics rescued 100 % of infected wt mice, and no deaths were observed. Likewise, Asm-deficient mice under antibiotic intervention were also completely protected from sepsis-induced lethality (Fig. 8).

Next, we determined the number of bacteria in organs of the mice. As shown above (Fig. 7a; supporting Fig. 8A), these results showed lower bacterial numbers in liver, spleen, and lung of wt mice compared to Asm-deficient mice 12 and 24 h after infection.

In summary, these data indicate that the inhibition of lung edema in Asm-deficient or amitriptyline-treated mice together with a sufficient antibiotic treatment, which reduces the number of bacteria, but does not alter the effect of bacterial toxins released during the infection or even after antibiotic treatment, is able to completely protect from lethality of *S. aureus* sepsis.

## Discussion

The results of the present study demonstrate that genetic deficiency or pharmacologic inhibition of the Asm/ceramide system protects against lung edema induced by systemic *S. aureus* infection. A principal hallmark of lung edema is degradation of TJ proteins, the disruption of endothelial cell integrity, and a resultant increase in vascular permeability and myeloid cell trafficking [6, 7]. All of these events are blocked in vivo and in vitro by genetic deficiency of Asm or treatment with the Asm inhibitor amitriptyline.

Our findings indicate that infection of endothelial cells with *S. aureus* activates Asm and thereby triggers the release of ceramide. Asm leads to the production of superoxide by endothelial cells. On the other hand, Asm activation is reduced by antioxidants, a result suggesting a positive feedback loop and a vicious cycle of *S. aureus*–induced Asm activation and superoxide release. This finding is similar to the results of previous studies showing a positive feedback loop between the Asm and superoxide after infection of macrophages with *P. aeruginosa* [30]. Recent studies showed that the translocation of lysosomal V1 H^+^-ATPase to the plasma membrane in coronary artery endothelial cells serves as a proton pump that contributes to the formation of small acidic regions at the cell surface. This shift in pH facilitates the activation of the Asm/ceramide system and leads to the formation of redox signalosomes, in which ceramide-enriched membrane domains are associated with the enhanced NADPH oxidase–mediated production of O_2_^-.^ [31, 32]. A similar mechanism may apply to the activation of the Asm by *S. aureus* on the cell surface. Recent studies revealed that superoxide can directly downregulate TJ proteins and indirectly activate matrix metalloproteinases (MMPs) that contribute to disrupt the integrity of endothelial cell layers. Moreover, superoxide directly activates several inflammatory cytokines, which in turn activate MMPs [33–35].

*S. aureus* is a leading cause of septic infections, and *S. aureus*–induced sepsis is one of the most serious infections acquired in hospitals or in the community. However, even with the use of appropriate antibiotics, fatal lung edema often develops [27–29]. Interestingly, our studies with wt and Asm-deficient mice seem to mimic the situation in hospitals showing a high lethality in septic *S. aureus* infections even if adequately treated with antibiotics [27–29].

Our findings demonstrate that treating mice with amitriptyline 1 or 2 h after infection reduces *S. aureus*–induced pulmonary edema and also inhibits myeloid cell trafficking and the degradation of TJ proteins. The reduced capacity of mice treated with amitriptyline or Asm-deficient mice to kill *S. aureus* is consistent with the previous notion that myeloid cells lacking Asm are unable to cluster and activate NADPH oxidases resulting in a defect of the production of superoxide and a reduced killing of pathogens. Finally, amitriptyline-treated or Asm-deficient mice died by the inability to eliminate the bacteria. In contrast, antibiotics kill the bacteria, but did not reduce lung edema. Thus, the combination of amitriptyline and antibiotics combines the advantages of inhibiting lung edema and eliminating systemic bacteria, protecting mice from lethality.

These findings also suggest that the Asm is required to mediate the effects of at least some *S. aureus* strains. *S. aureus* expresses multiple toxins, such as alpha-toxin, Panton-Valentine leukocidin, and enterotoxin B and A, which cause membrane damage, infiltration of myeloid cells and macrophages, cytokine production, and increased vascular permeability resulting in severe pulmonary edema and lung injury [36–39]. Both of the *S. aureus* strains used in our study produce several hemolysins. The pore-forming alpha-toxin, which is one of the best-characterized virulence factors of *S. aureus*, is involved in the pathogenesis of skin infections, pneumonia, and sepsis, including those caused by MRSA. At present, it is unknown whether purified alpha-toxin activates the Asm in human and mouse monocytic cells. Recently, it was reported that the binding of alpha-toxin to its eukaryotic receptor A-disintegrin and metalloprotease 10 (ADAM10) leads to the up-regulation of ADAM10 activity, which is required for alpha-toxin-induced cytotoxicity [40–42]. Increased ADAM10 activity in epithelial and endothelial cells disrupts the cell barrier function, and this disruption contributes to the pathogenesis of lethal lung edema. However, it is unknown whether the Asm and ADAM10 function in the same signaling cascade or are independent pathways that are both required for the cellular effects of alpha-toxin.

Here, we demonstrated that the combination of amitriptyline and antibiotics effectively protects mice from lung edema and bacteremia during sepsis. Amitriptyline is a well-known antidepressant that has been widely used in clinical practice for more than 50 years and is associated with only mild adverse effects at therapeutic doses. Thus, inhibition of the Asm/ceramide system in combination with antibiotics could be a novel approach to treat severe systemic and often lethal infections and to inhibit lung injury in patients with incipient sepsis.

## Electronic supplementary material

Below is the link to the electronic supplementary material.

ESM 1 (PDF 8449 kb)
