# Supplementary material for: Acid sphingomyelinase inhibition protects mice from lung edema and lethal Staphylococcus aureus sepsis
Source: J Mol Med (Berl). 2015 Jan 25;93(6):675–89. doi: 10.1007/s00109-014-1246-y (PMC4432103; doi:10.1007/s00109-014-1246-y)
Supplement: Supplementary file 1 — (PDF 8449 kb) [file 109_2014_1246_MOESM1_ESM.pdf]

J Mol Med 2015

Acid sphingomyelinase inhibition protects mice from lung edema and lethal *Staphylococcus aureus* sepsis

Huiming Peng, Cao Li, Stephanie Kadow, Brian D. Henry, Jörg Steinmann, Katrin Anne Becker, Andrea Riehle, Natalie Beckmann, Barbara Wilker, Pin-Lan Li, Timothy Pritts, Michael J. Edwards, Yang Zhang, Erich Gulbins, Heike Grassmé

SUPPORTING INFORMATION

Supporting Figure 1:

a

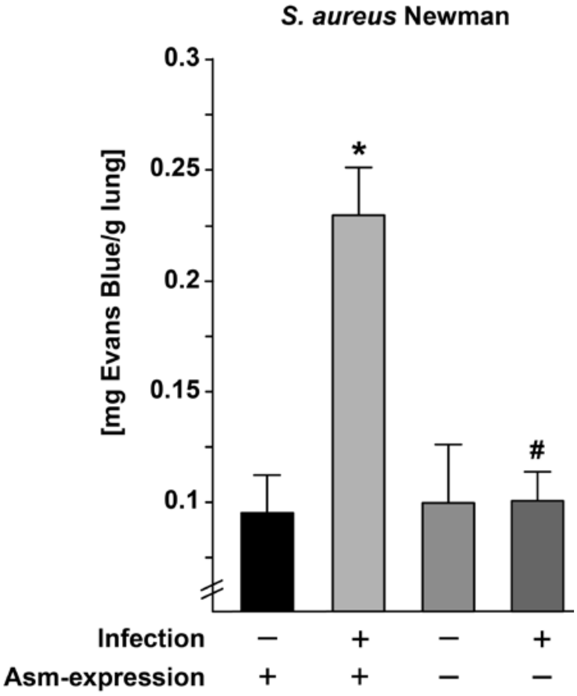

b

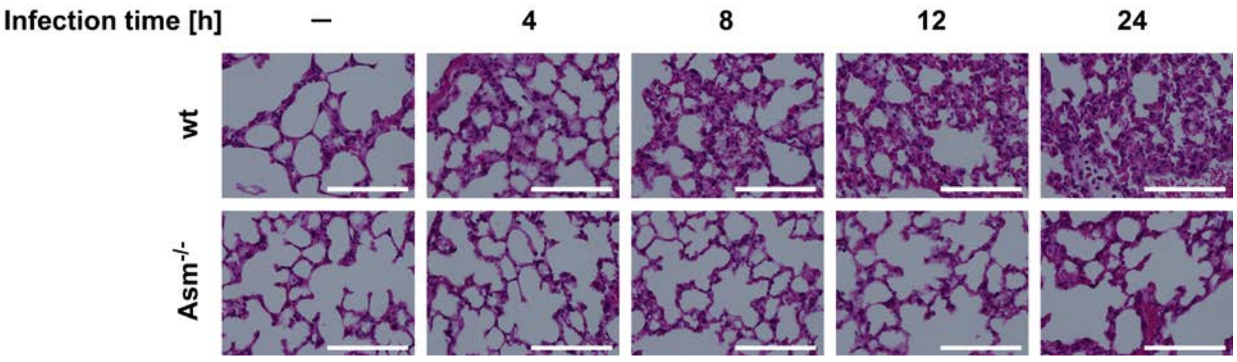

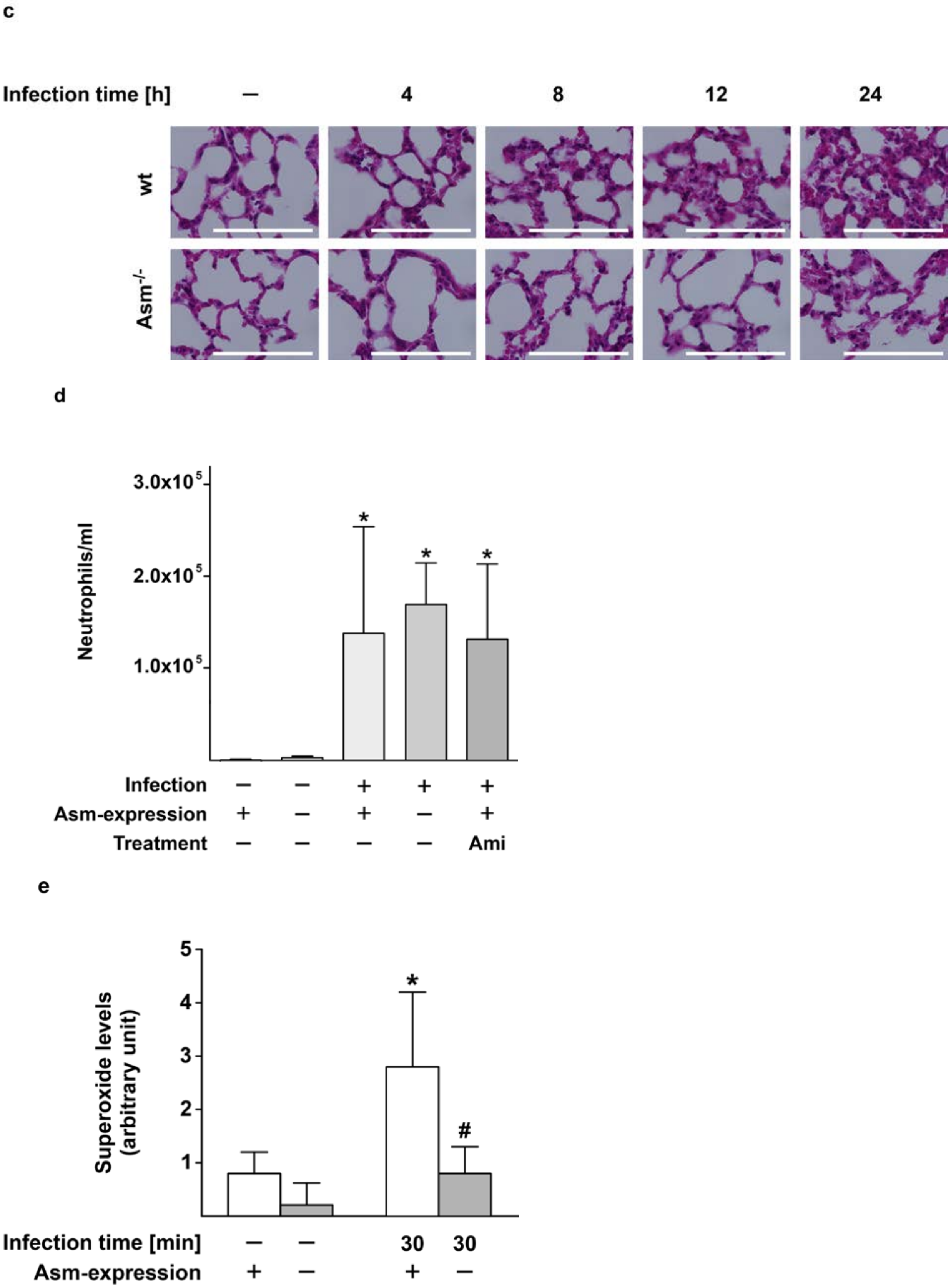

Figure 1. Infection with *S. aureus* results in edematous changes in lungs of wt but not Asm-deficient mice

(**A**) Infection of mice with *S. aureus* Newman resulted in lung edema determined by extravasation of Evans Blue. Mice were infected for 12 hrs, the lungs flushed with PBS,

removed and Evans Blue extravasation was determined. Data are presented as mean ± SD of at least three independent experiments. *, significant differences between uninfected and infected samples; #, significant differences between wildtype (+) and Asm-deficient (-) mice (P<0.05; *t*-test).

(B,C) Wild-type (wt) and acid sphingomyelinase (Asm)-deficient mice (Asm$^{-/-}$) were infected intravenously with 5x10^{6} colony-forming units (CFU) of a clinical *S. aureus* isolate for the indicated time points. Mice were sacrified, lungs were removed and stained for H&E with high magnification. All images are representative of three independent experiments. (Scale bar is 100 µm). (**D,E**) The neutrophil population from wt, Asm$^{-/-}$, or amitriptyline (Ami) treated mice in peritoneal lavage was collected and either stained with FITC-labeled GR1-antibodies and analyzed by FACS (**D**) or examined for superoxide production **(E)**. *, significant differences between uninfected and infected samples; #, significant differences between treated and untreated or wt amd Asm-deficient mice (P<0.05, *t*-test).

Supporting Figure 2:

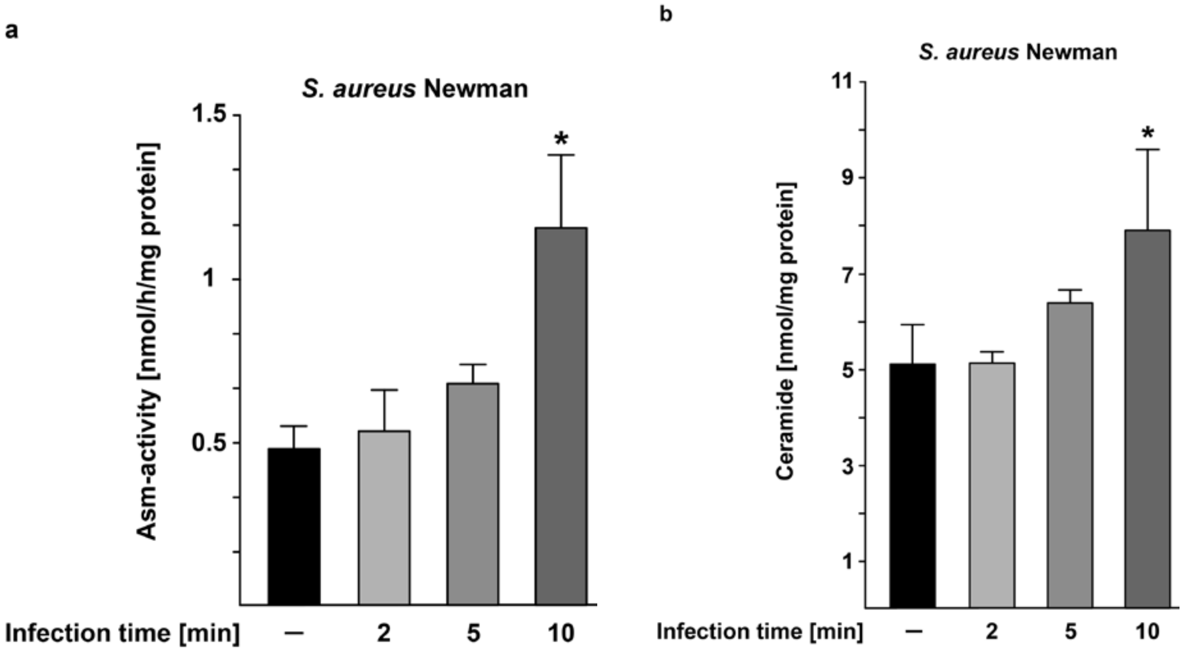

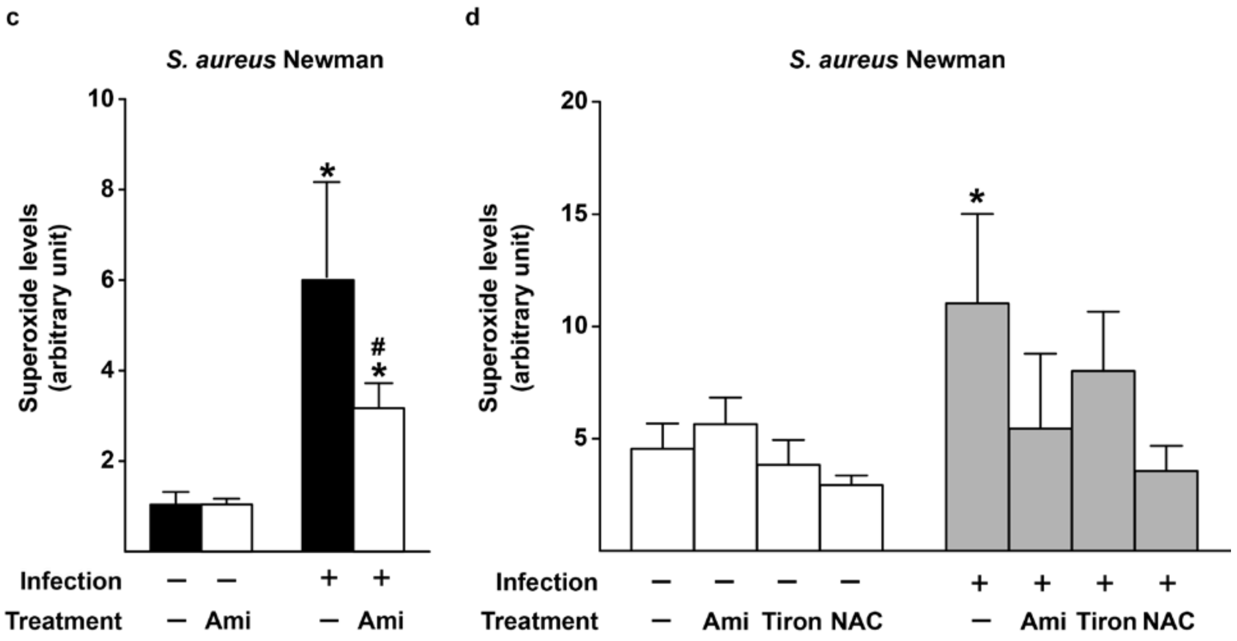

Figure 2. Infection of endothelial cells with the *S. aureus* strain Newman activates the acid sphingomyelinase/ceramide system and triggers the release of superoxide

EOMA-cells were infected with *S. aureus* Newman (MOI 200:1) for the indicated time periods. Asm activity **(A)** and ceramide concentrations **(B)** were measured 0, 1, 2, 5, or 10 min after infection. For the detection of superoxide **(C)**, cells were pre-incubated with amitriptyline (Ami) 20 min before infection for the indicated time or left untreated and/or uninfected. The production of superoxide was quantified by electron spin resonance. Relative O_2^- levels were used to indicate superoxide accumulation. Shown are means of arbitrary units ± SD from at least three independent experiments. *, significant differences between uninfected and infected samples; #, significant differences between treated and untreated samples (P<0.05; *t*-test).

Supporting Figure 3:

a

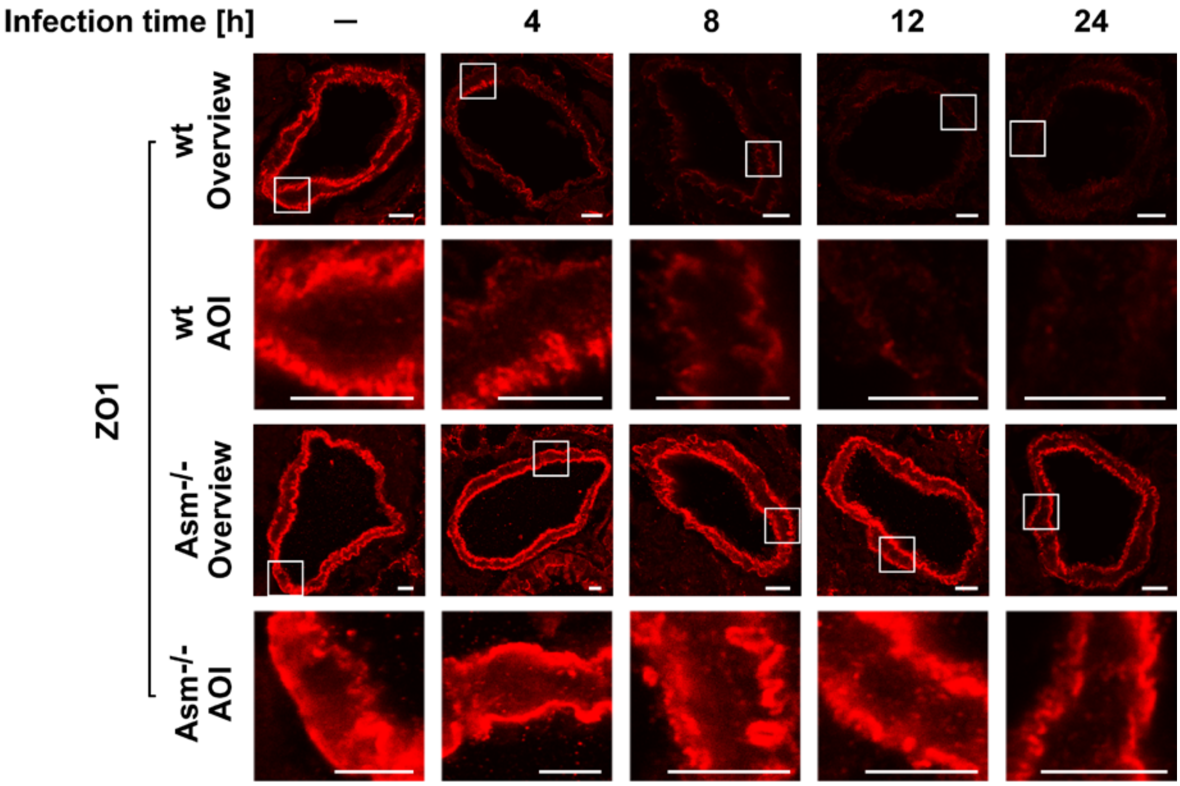

b

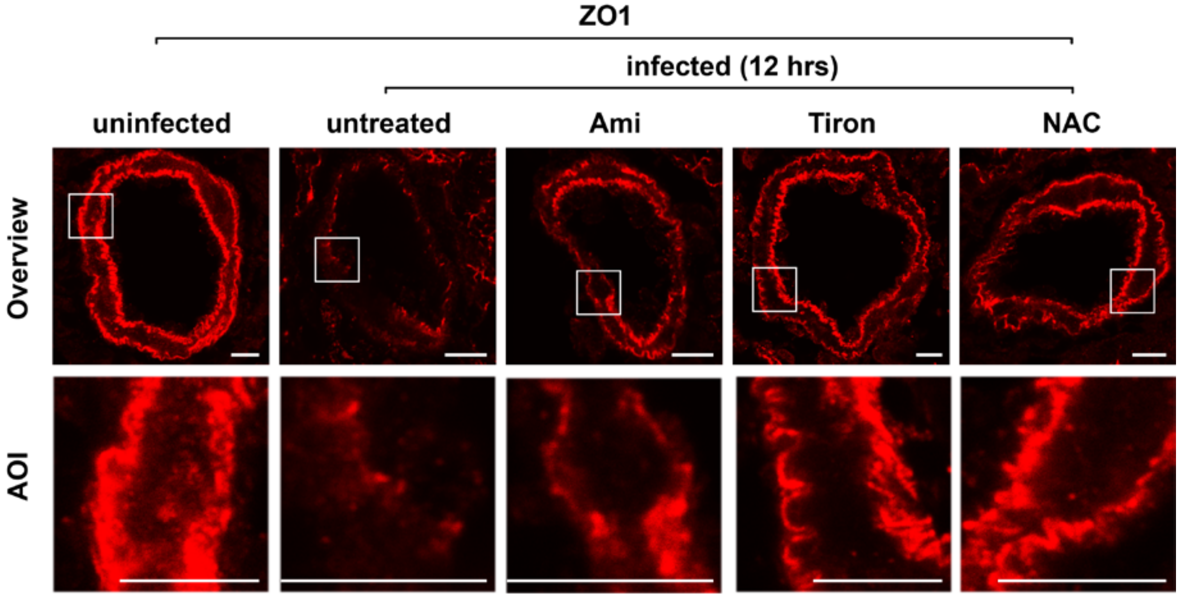

c

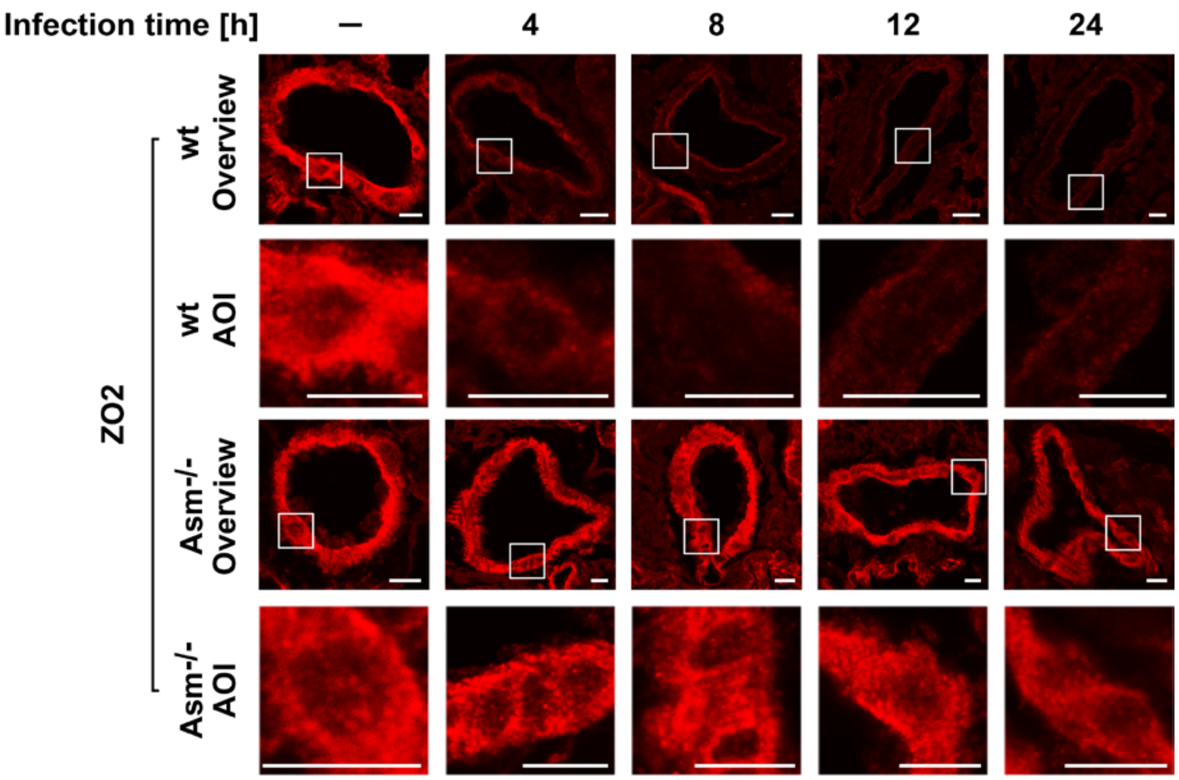
Infection time [h]
–
4
8
12
24
ZO2
wt Overview
wt AOI
Asm-/- Overview
Asm-/- AOI

d

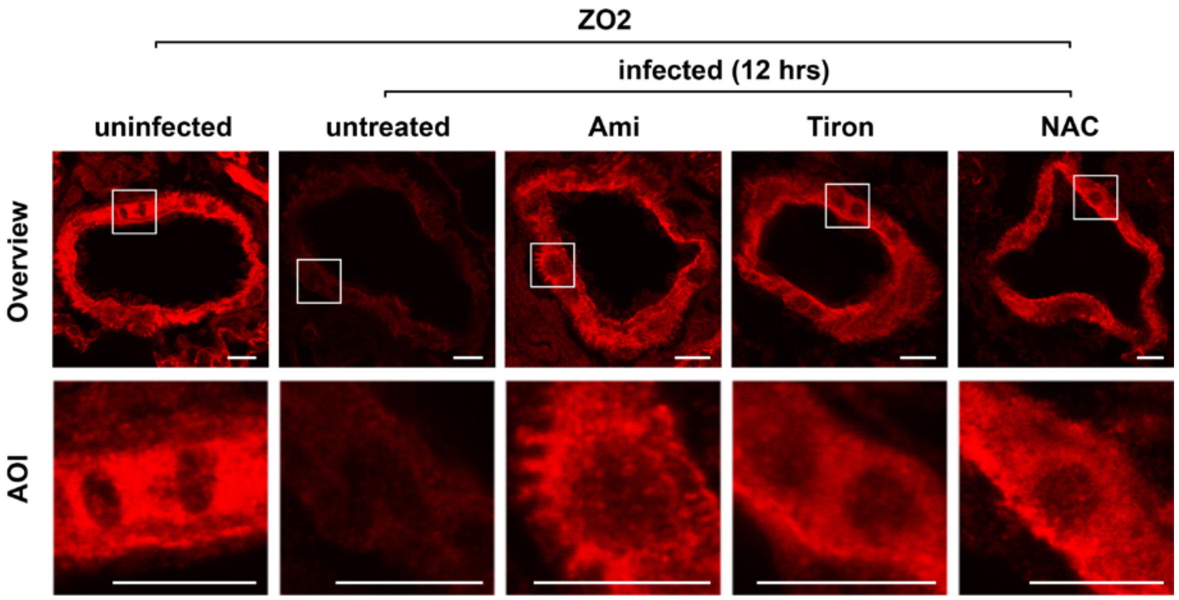
ZO2
infected (12 hrs)
uninfected
untreated
Ami
Tiron
NAC
Overview
AOI

e

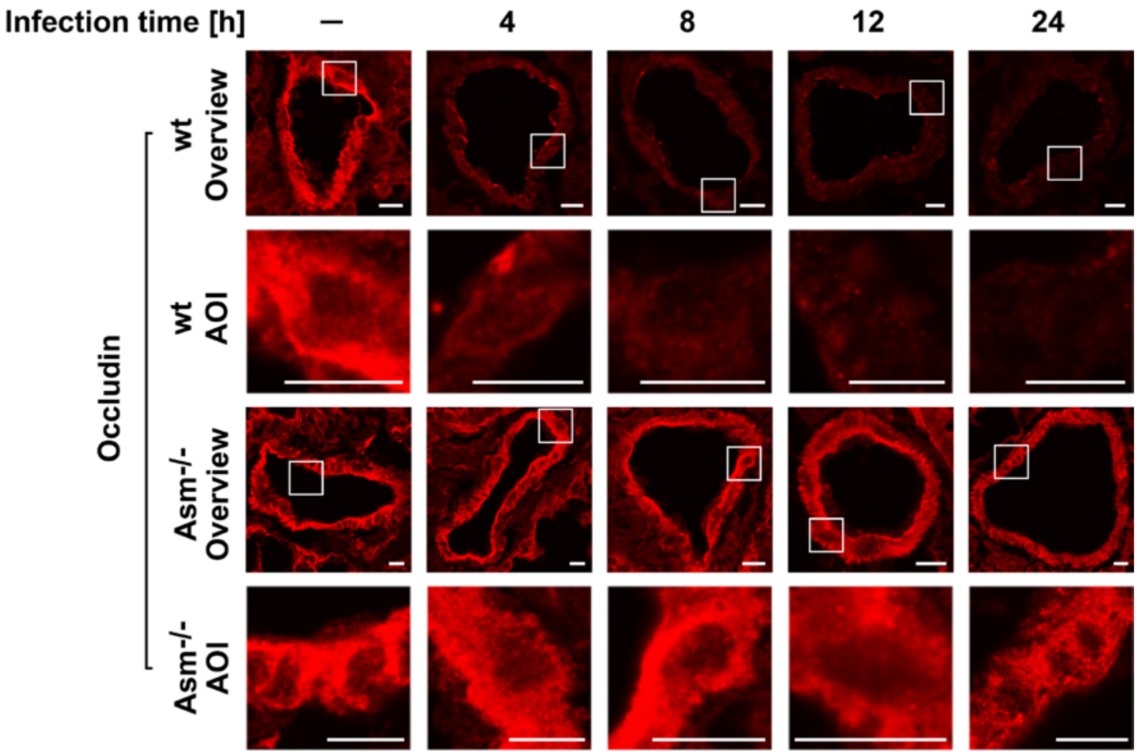
Infection time [h]
—
4
8
12
24
Occludin
wt Overview
wt AOI
Asm-/- Overview
Asm-/- AOI

f

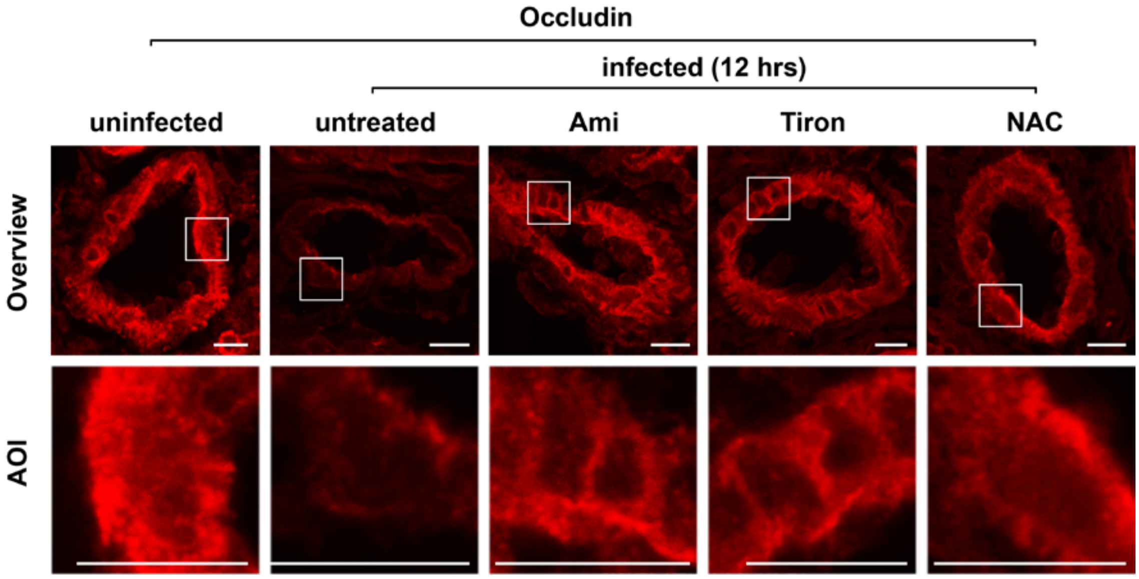
Occludin
infected (12 hrs)
uninfected
untreated
Ami
Tiron
NAC
Overview
AOI

g

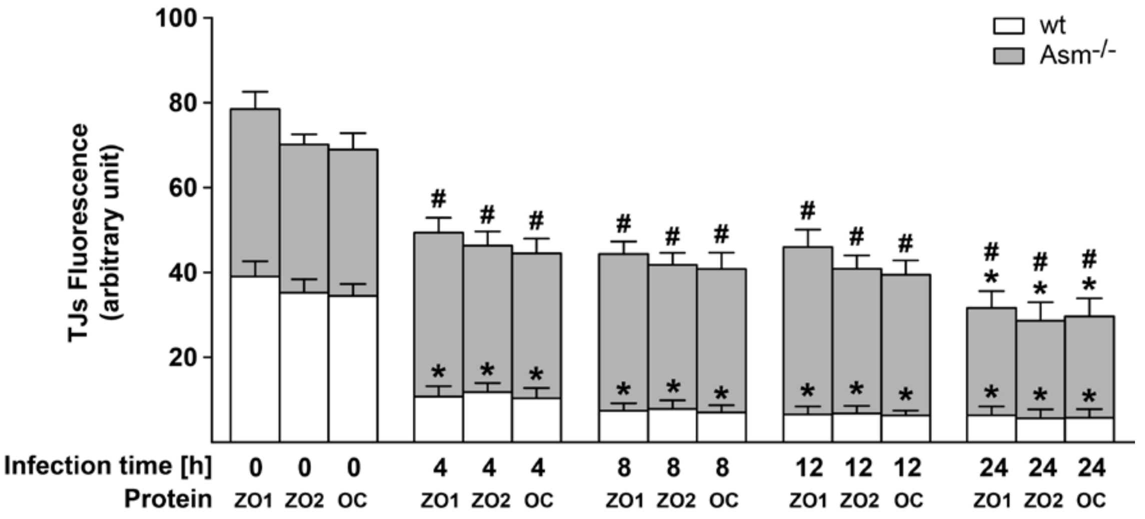

h

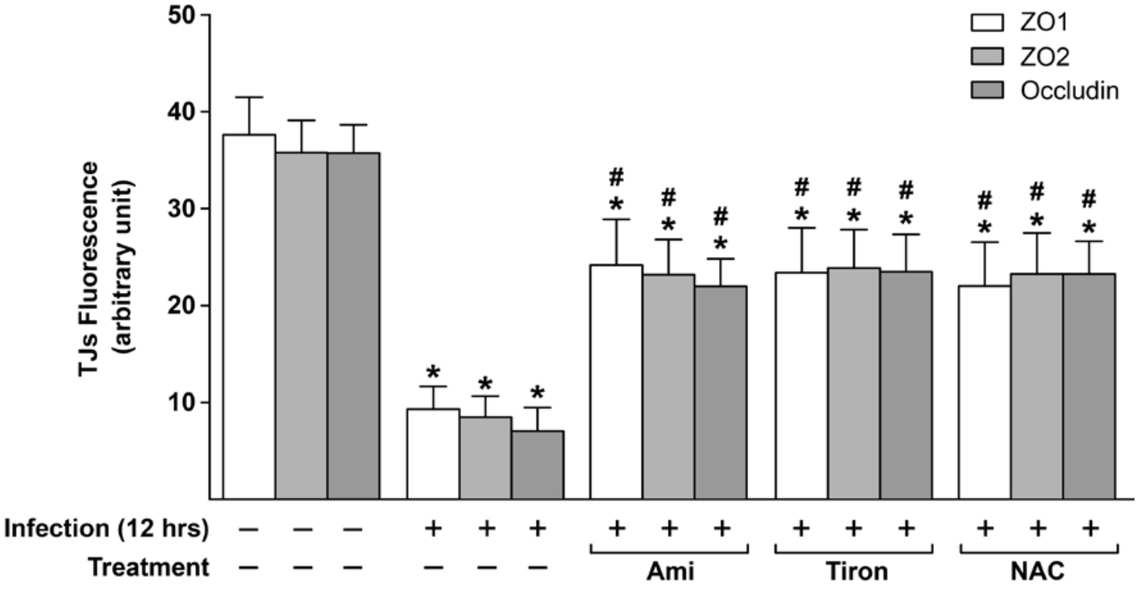

Figure 3,4. The disruption of tight junction proteins after infection is reduced by inhibiting activation of Asm and production of superoxide

Fig. 3: Wild-type (wt) and Asm-deficient (Asm$^{-/-}$) mice were left uninfected or were infected with the clinical *S. aureus* isolate for the indicated time points. Wt mice were pretreated before infection (12 hrs) with amitriptyline (Ami) (10 mg/kg) or the antioxidants Tiron (100 mg/kg) or N-Acetylcysteine (NAC) (100 mg/kg), or left untreated and/or uninfected. Lung sections were stained with Cy3-coupled antibodies against ZO1 **(A,B)**, ZO2 **(C,D)** or occludin (OC) **(E,F).** We scored tight junctions (TJs) fluorescence via Photoshop (10 pictures per lung) **(G,H)** *, significant differences between uninfected and infected samples; #, significant differences between wt and Asm-deficient or untreated and treated mice, respectively (P<0.05; *t*-test).

Supporting Figure 4:

a

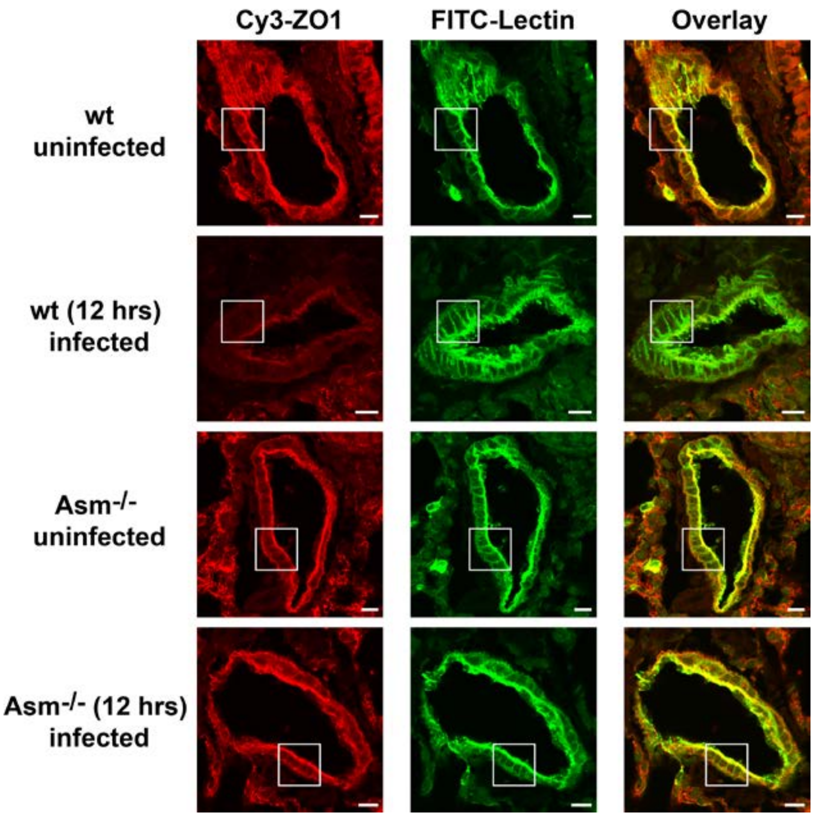

b

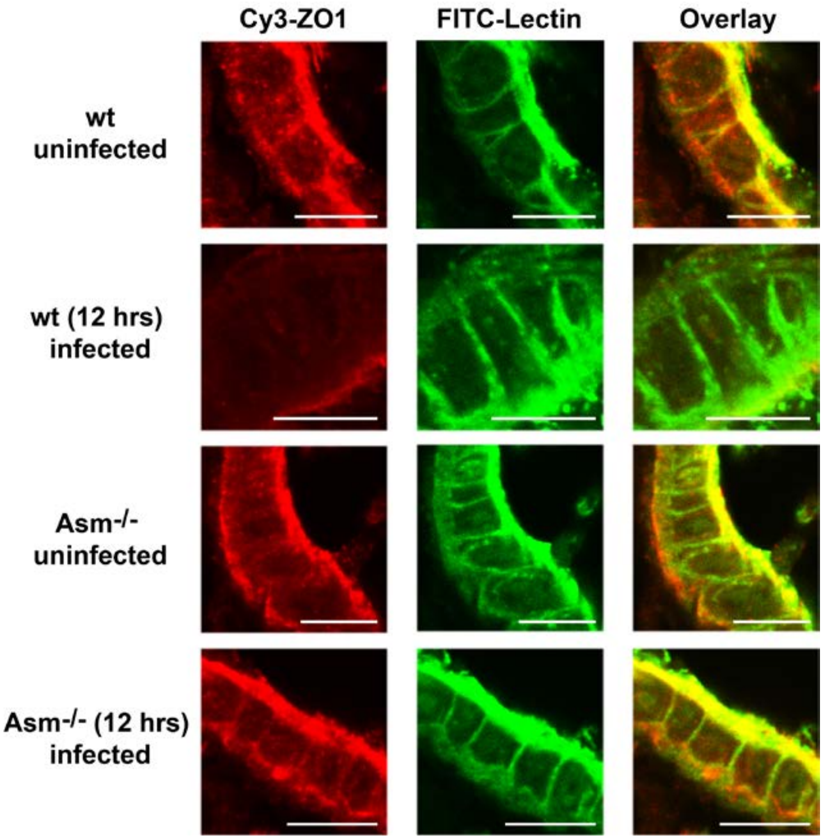

c

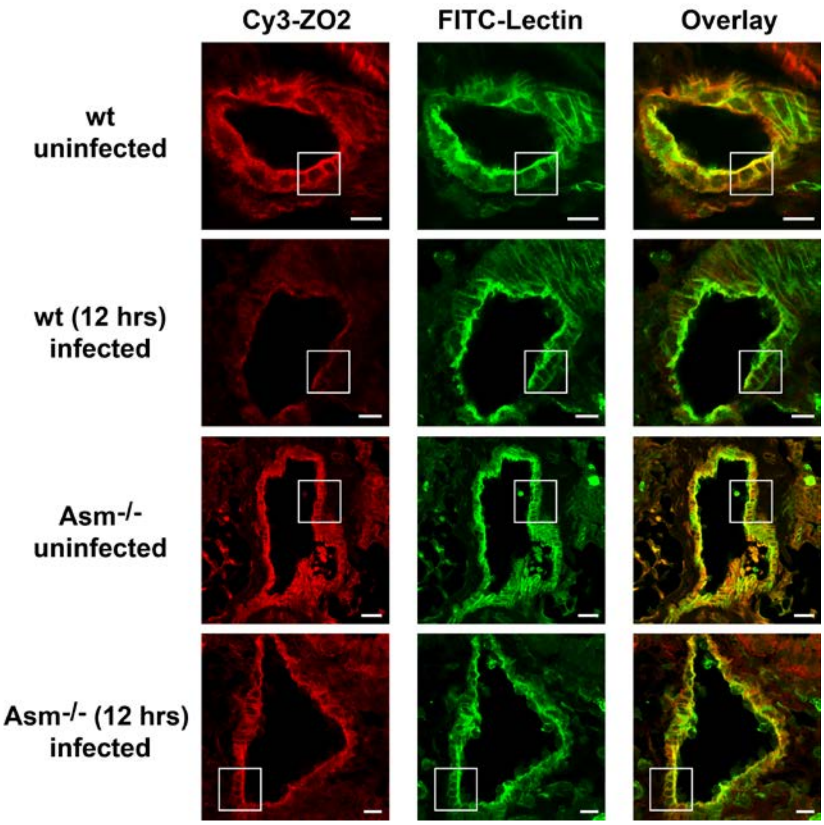
Cy3-ZO2
FITC-Lectin
Overlay
wt
uninfected
wt (12 hrs)
infected
Asm-/-
uninfected
Asm-/- (12 hrs)
infected

d

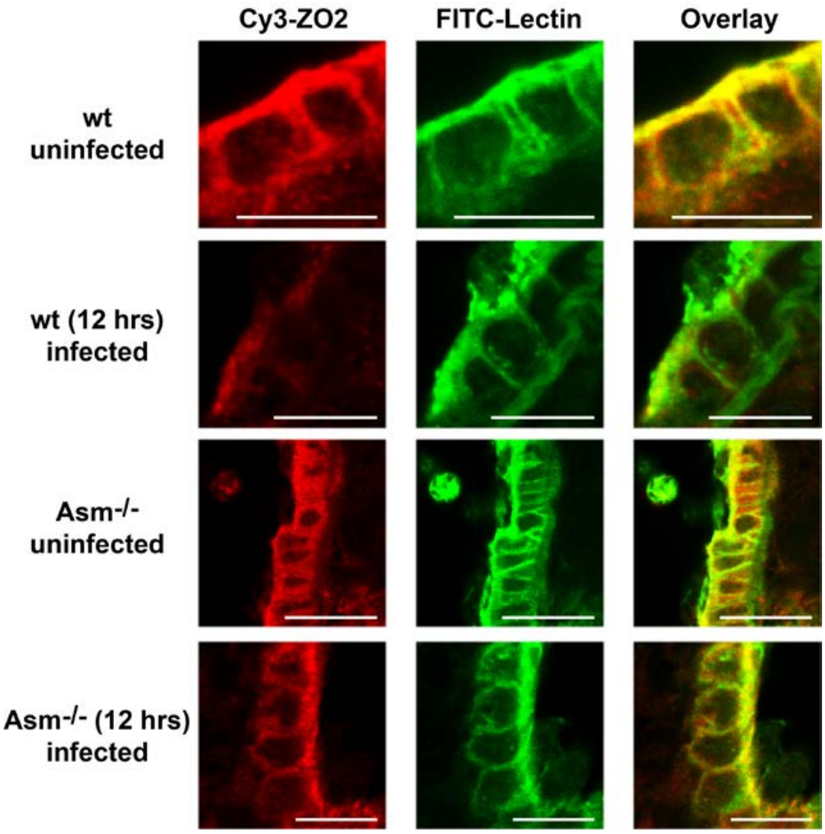
Cy3-ZO2
FITC-Lectin
Overlay
wt
uninfected
wt (12 hrs)
infected
Asm-/-
uninfected
Asm-/- (12 hrs)
infected

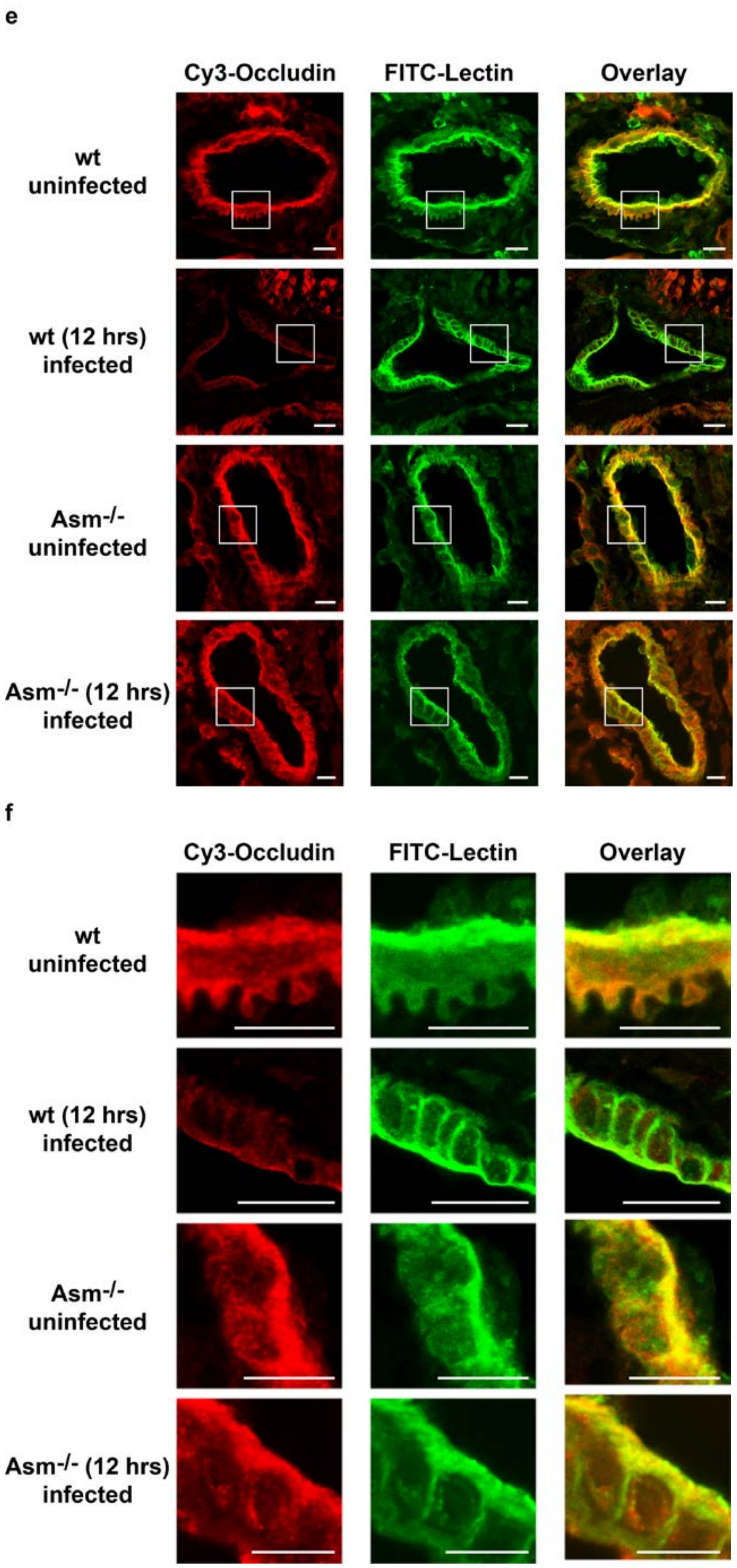

Fig. 4: Endothelial cells were costained for TJs ZO1, ZO2 or occludin and the endothelial cell marker FITC-lectin **(A-F)** and analyzed by confocal microscopy. Representative images from

three independent experiments are shown (original image and an area of interest [AOI]). Scale bar is 10 μm.

Supporting Figure 5:

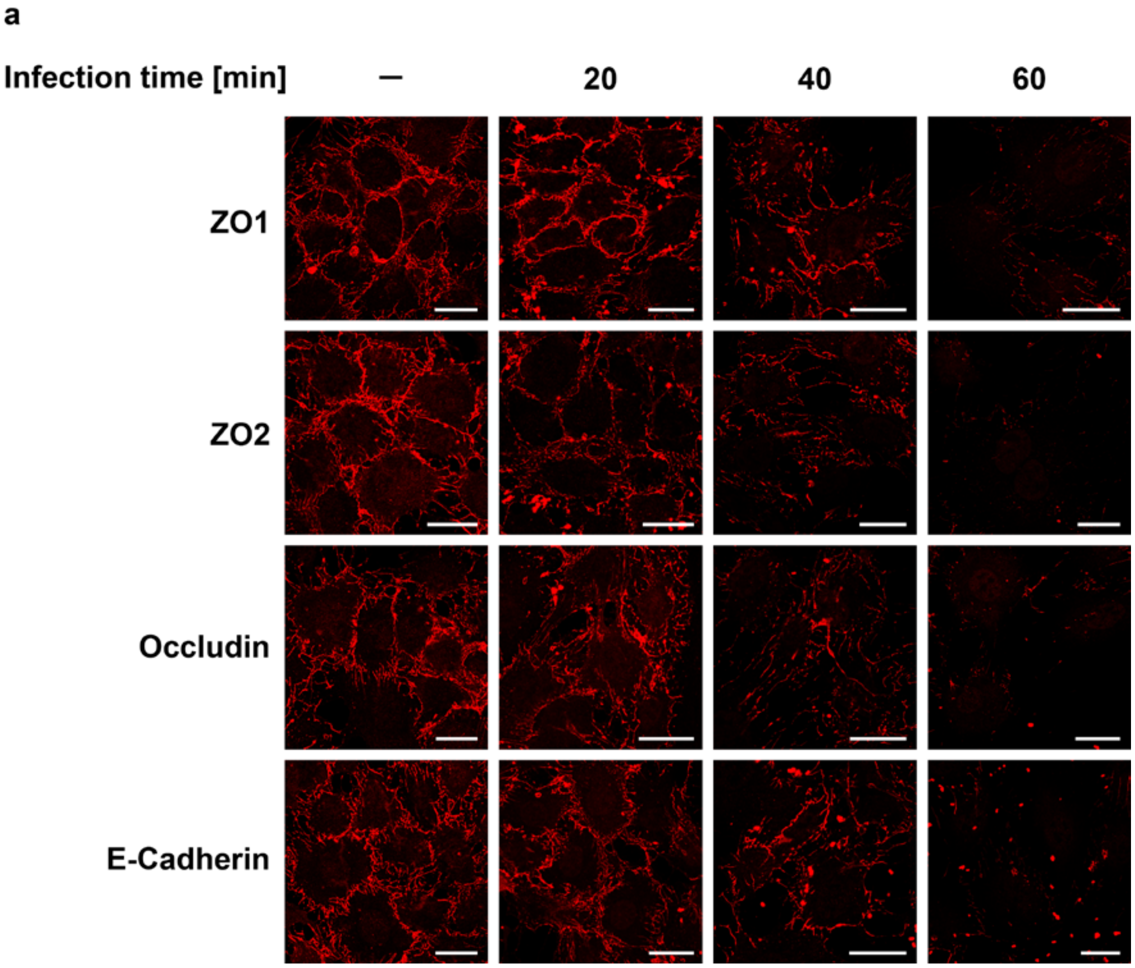

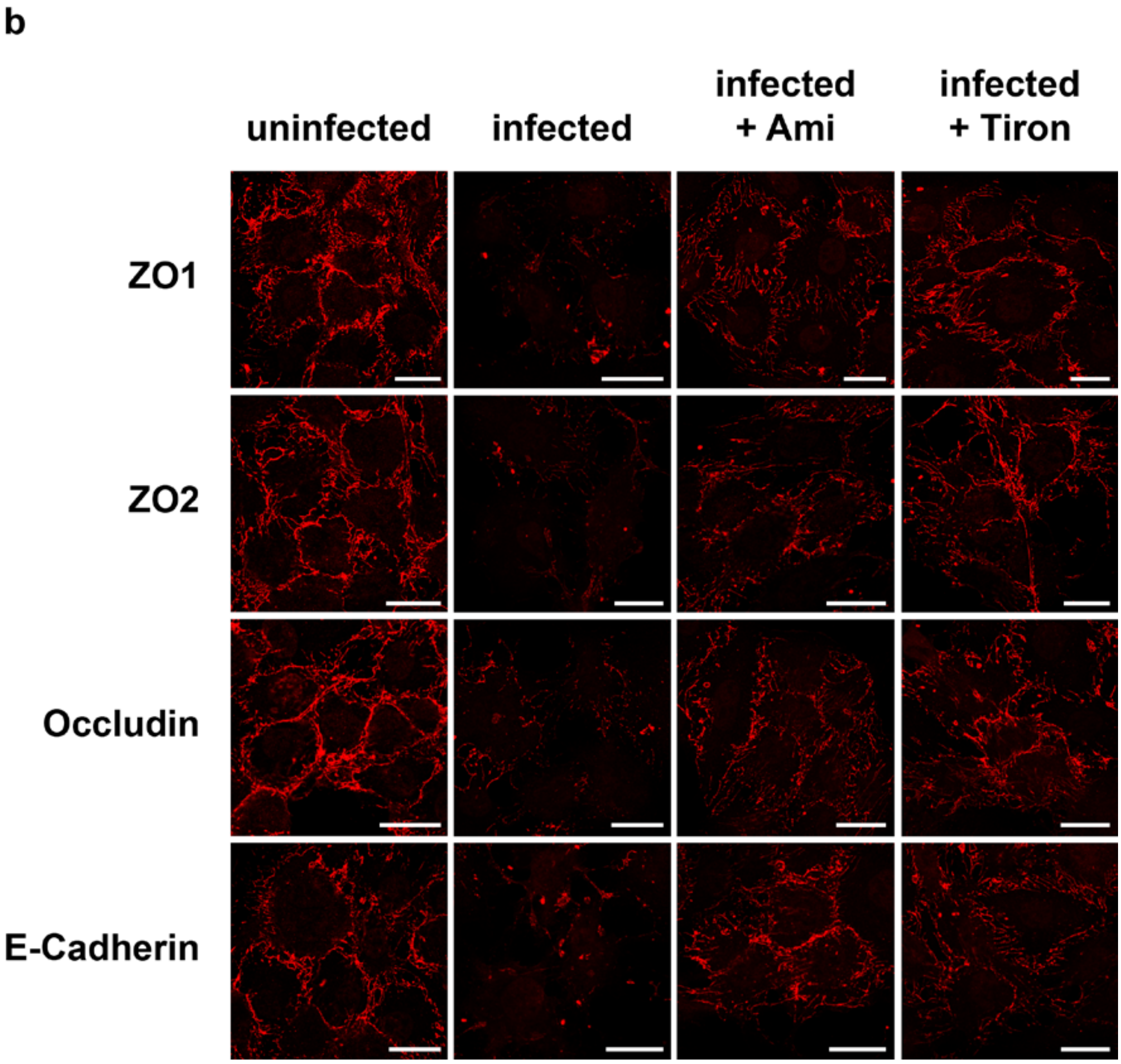

Figure 5. Disruption of tight junctions after infection of EOMA cells is inhibited by amitriptyline or Tiron

Endothelial cells were infected with *S. aureus* at a MOI 200:1. The disruption of tight junctions (TJs) ZO1, ZO2, occludin or E-cadherin after different infection time **(A)** and after treatment with inhibitors (60 min of infection) **(B)** was determined by staining with Cy3-labeled antibodies and analyzed by confocal microscopy. The presented pictures are representative of the results of at least three independent experiments. Scale bar is 25 μm.

Supporting Figure 6:

a

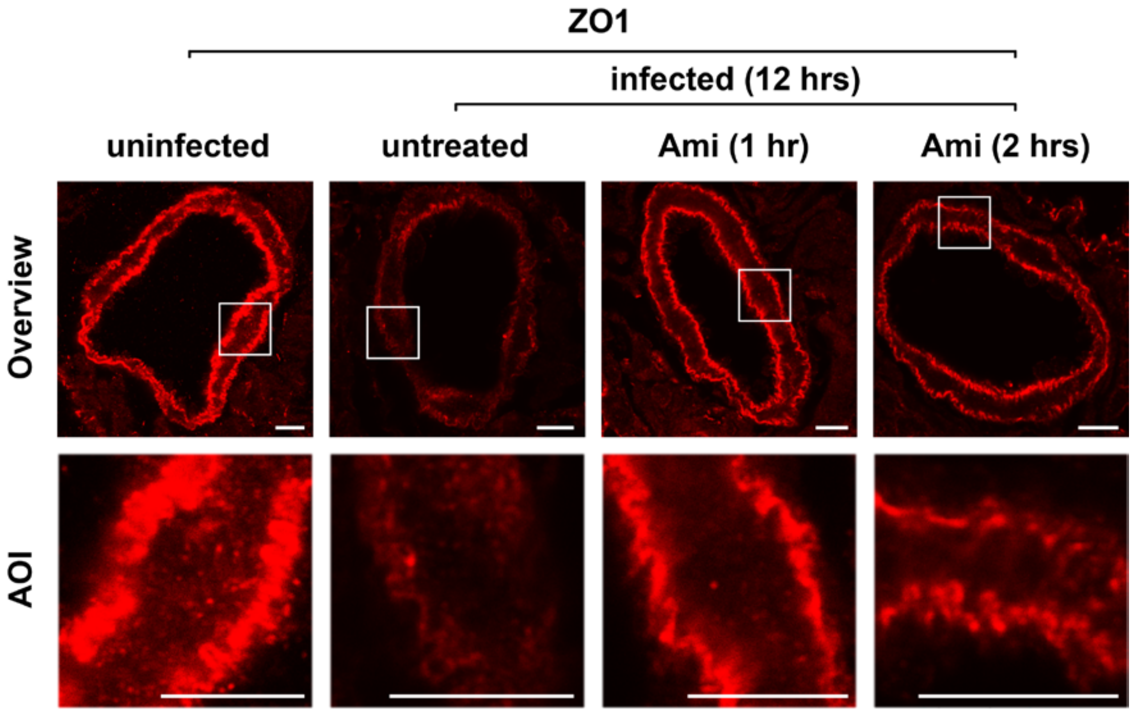

b

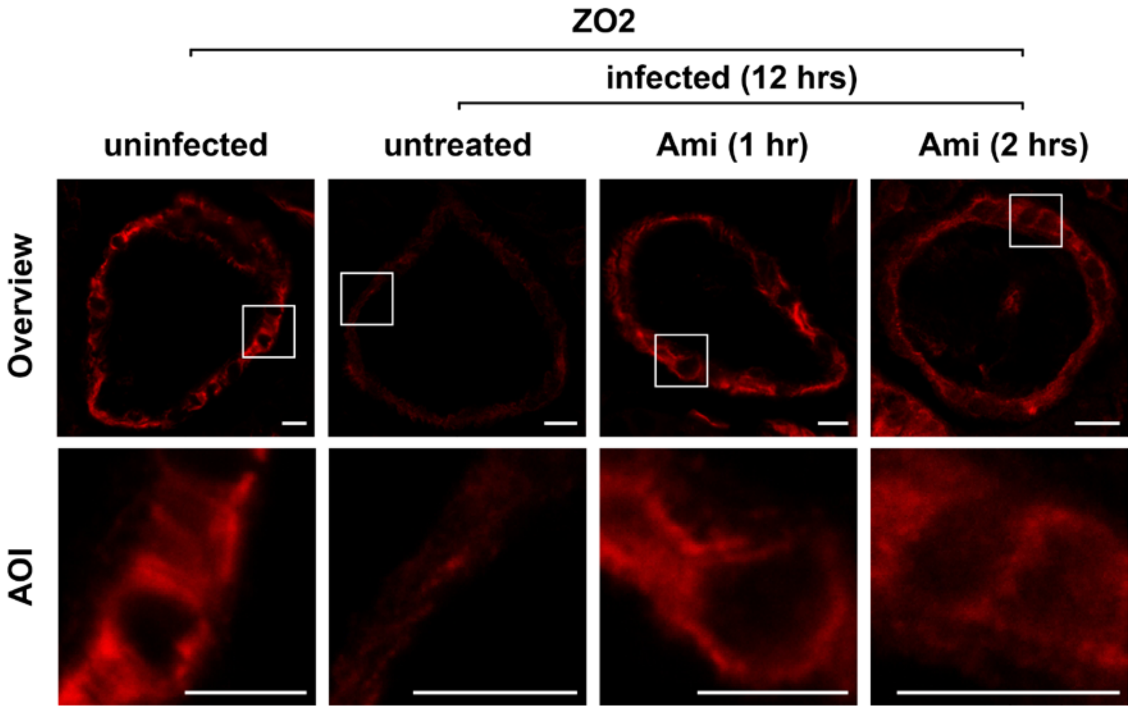

d

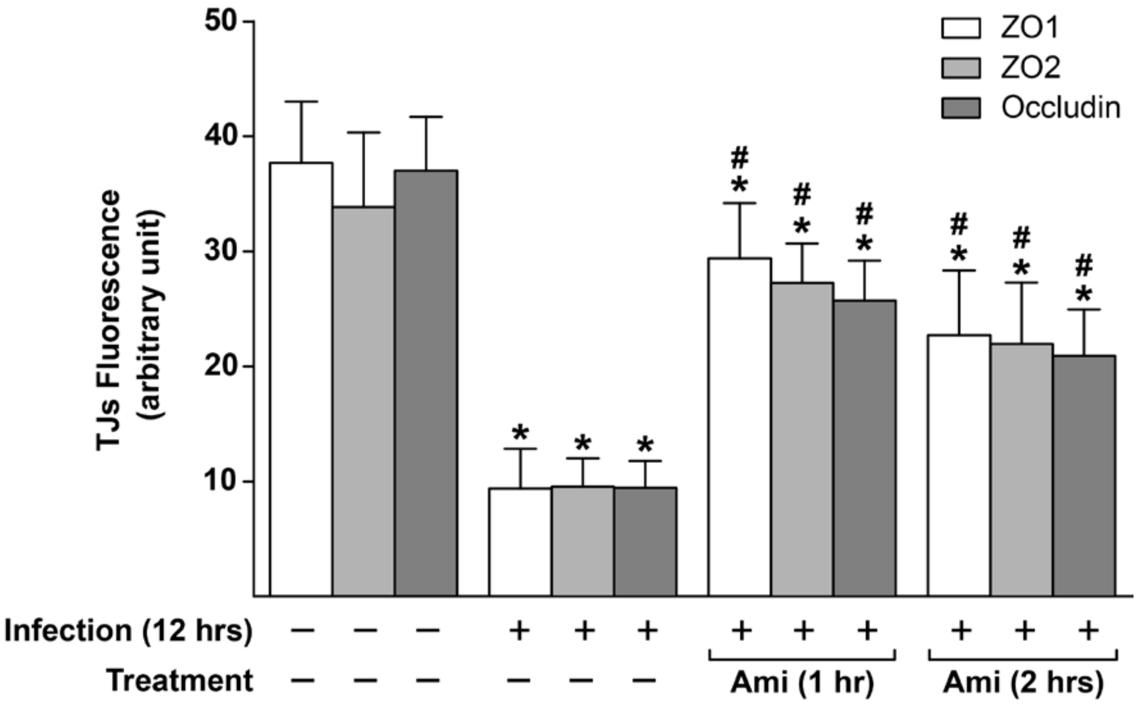

Figure 6. Amitriptyline treatment reduces degradation of tight junction proteins even if administered after infection

Wt mice were infected with the clinical *S. aureus* isolate and were treated 1 hr or 2 hrs after infection with i.p. injected amitriptyline (Ami). 12 hrs after infection, the mice were sacrificed. Lung sections were stained with Cy3-labeled antibodies against ZO1 **(A)**, ZO2 **(B)** or occludin **(C)**. Images were obtained by confocal microscopy and are representative of three independent experiments. The original image and an area of interest (AO1) are shown. Scale bar is 10 µm. **(D)** Scoring of fluorescence intensity was performed using Photoshop (10 pictures per lung), *, significant differences between uninfected and infected samples; #, significant differences between untreated and treated samples, respectively (P<0.05; t-test).

Supporting Figure 7:

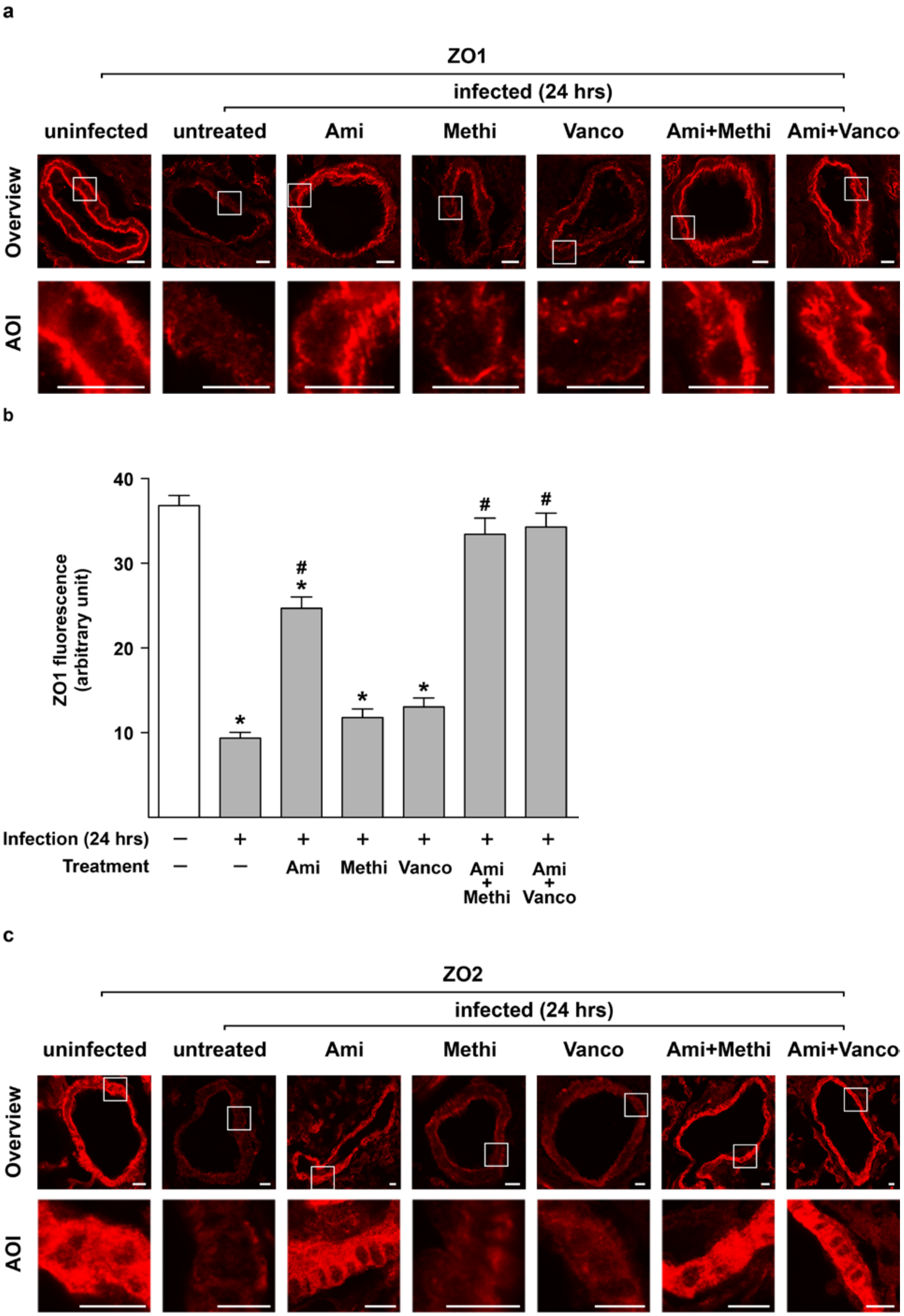

d

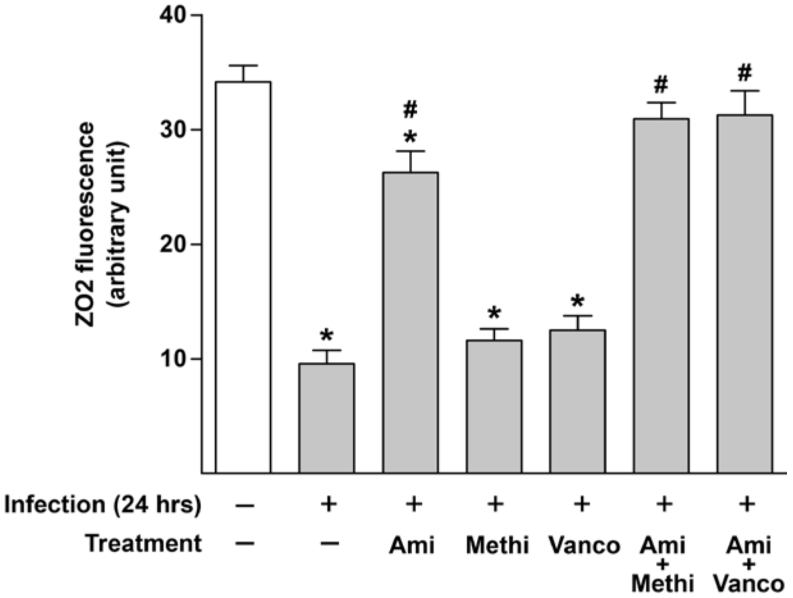
ZO2 fluorescence
(arbitrary unit)
40
30
20
10
#
*
#
#
Infection (24 hrs) – + + + + + +
Treatment – – Ami Methi Vanco Ami + Methi Ami + Vanco

e

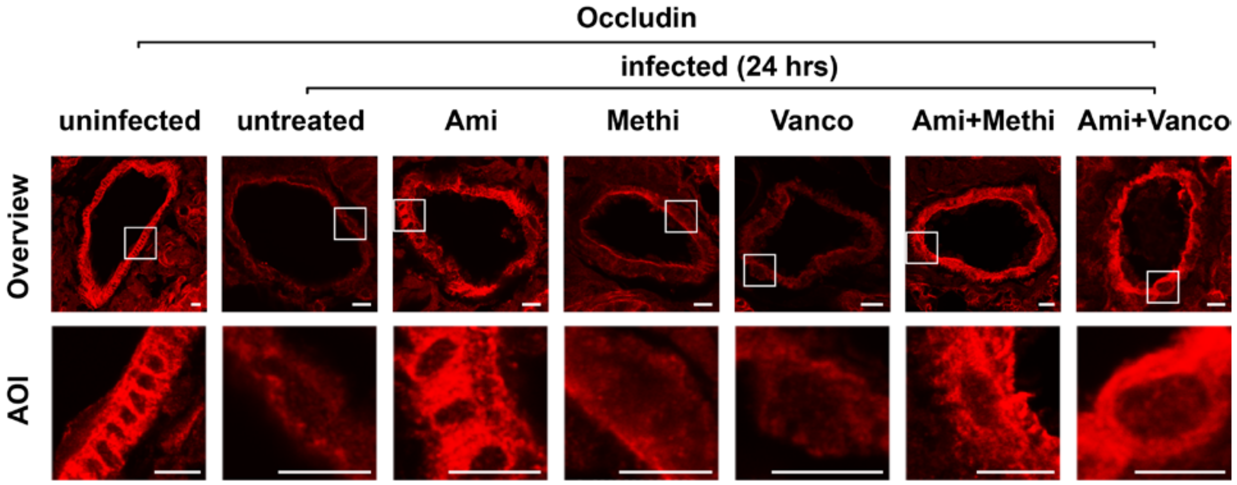
Occludin
infected (24 hrs)
uninfected
untreated
Ami
Methi
Vanco
Ami+Methi
Ami+Vanco
Overview
AOI

f

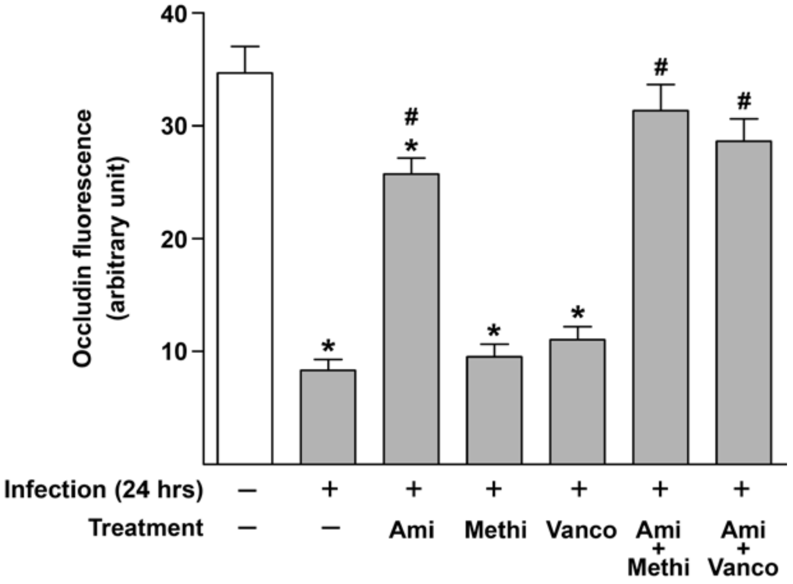
Occludin fluorescence
(arbitrary unit)
40
30
20
10
#
*
#
#
Infection (24 hrs) – + + + + + +
Treatment – – Ami Methi Vanco Ami + Methi Ami + Vanco

Figure 7. The combination of amitriptyline and antibiotics inhibits *S. aureus* induced breakdown of tight junction proteins

Wt mice were infected with a clinical isolate *S. aureus*. They were then left untreated; treated with an i.p. injection of amitriptyline (Ami) (16 mg/kg) 1 hr after infection; treated with either methicillin (Methi) or vancomycin (Vanco) (both 100 mg/kg) 1 hr and 9 hrs after infection; or treated with the combination of amitriptyline and methicillin or vancomycin. The mice were sacrificed 24 hrs after infection. Lung sections were stained with Cy3-labeled antibodies against ZO1 **(A),** ZO2 **(C)** or occludin **(E)** and confocal microscopy was performed (scale bar is 10 μm). Shown are representative images from three independent experiments. Tight junction (TJ)-flourescence intensity was scored using Photoshop (10 pictures per lung) **(B,D,F)**. Data are shown as mean ± SD, n = 3. *, significant differences between uninfected and infected samples; #, significant differences between treated and untreated samples (all $P<0.05$, *t*-test).

Supporting Figure 8:

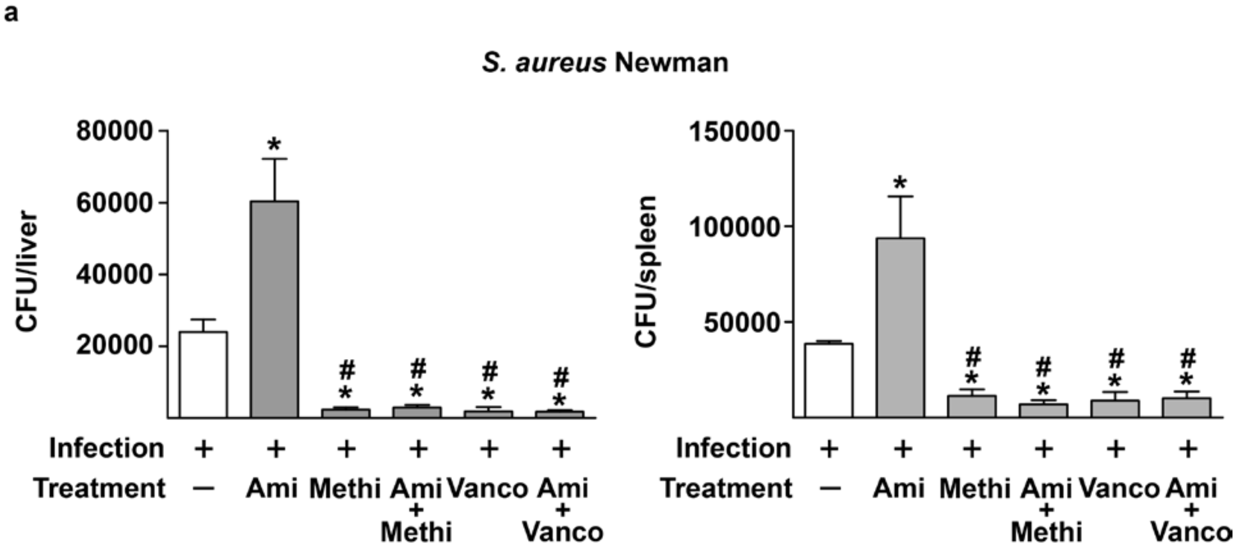

b

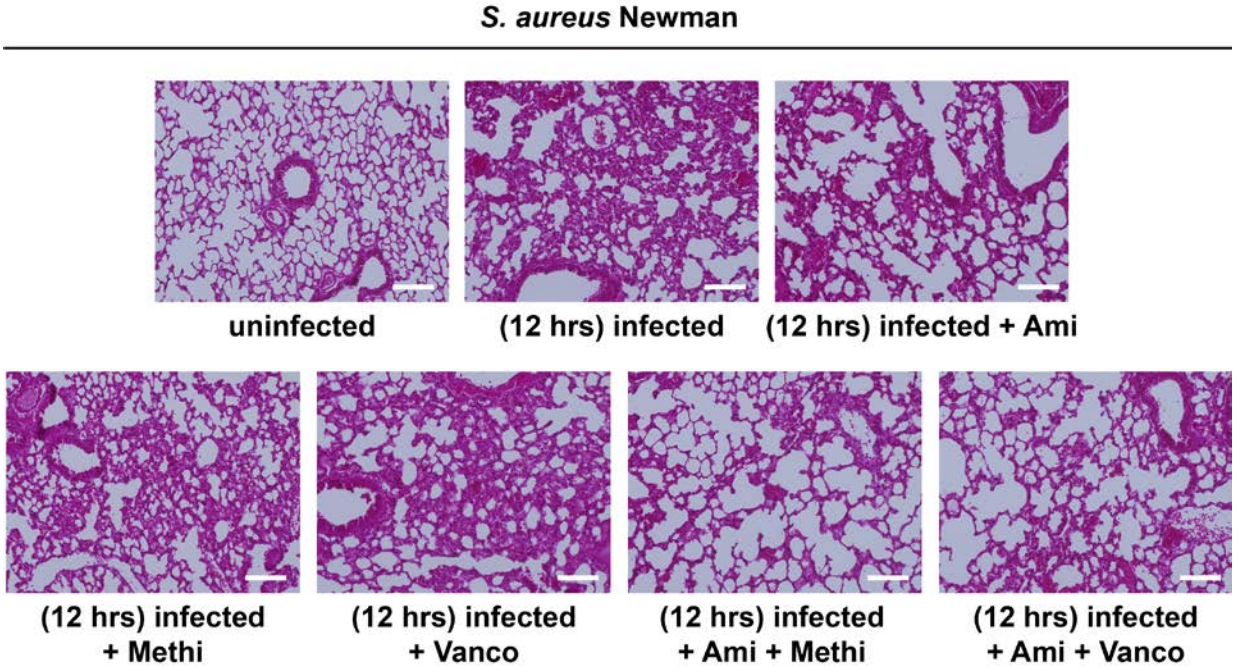
S. aureus Newman
uninfected
(12 hrs) infected
(12 hrs) infected + Ami
(12 hrs) infected + Methi
(12 hrs) infected + Vanco
(12 hrs) infected + Ami + Methi
(12 hrs) infected + Ami + Vanco

c

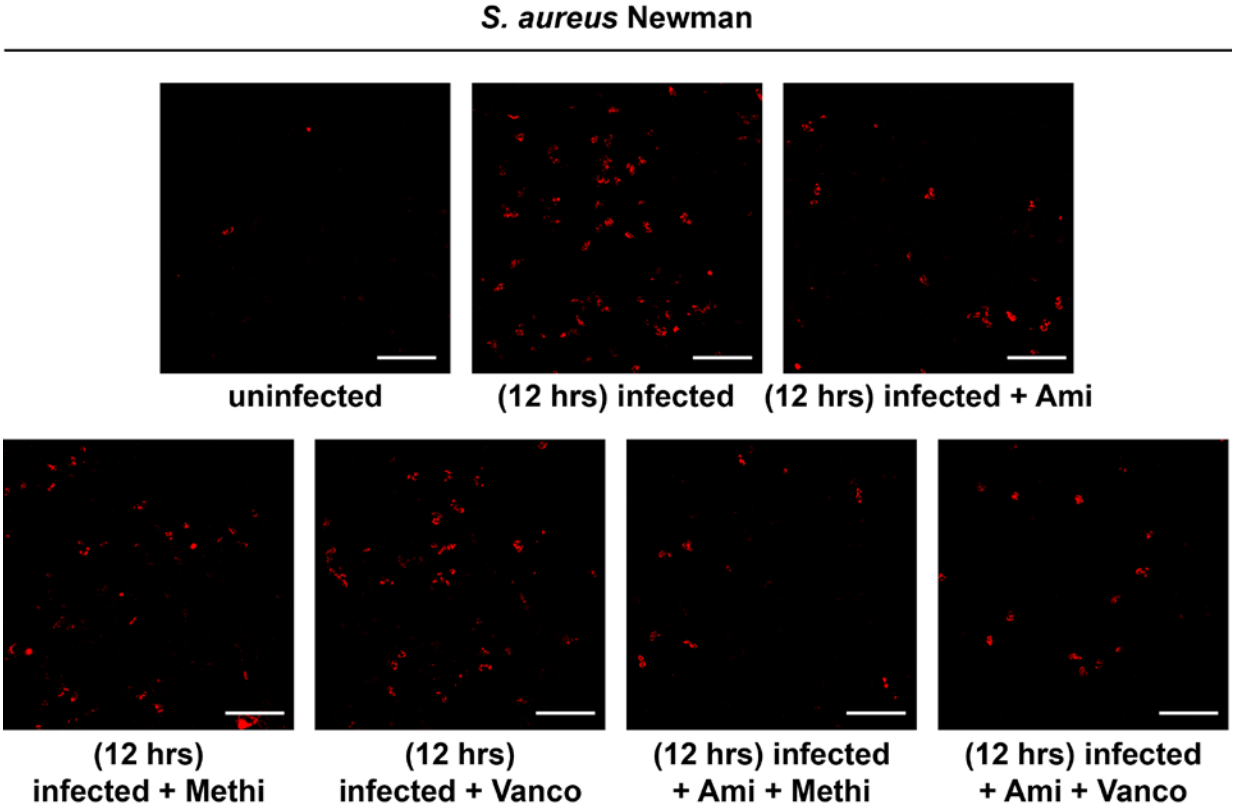
S. aureus Newman
uninfected
(12 hrs) infected
(12 hrs) infected + Ami
(12 hrs) infected + Methi
(12 hrs) infected + Vanco
(12 hrs) infected + Ami + Methi
(12 hrs) infected + Ami + Vanco

d

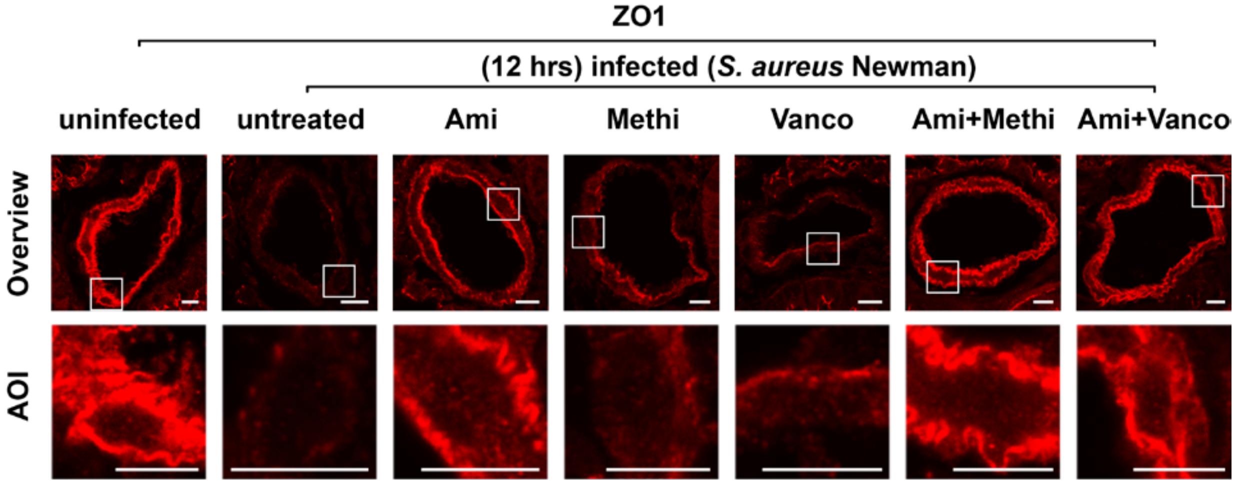
ZO1
(12 hrs) infected (S. aureus Newman)
uninfected
untreated
Ami
Methi
Vanco
Ami+Methi
Ami+Vanco
Overview
AOI

e

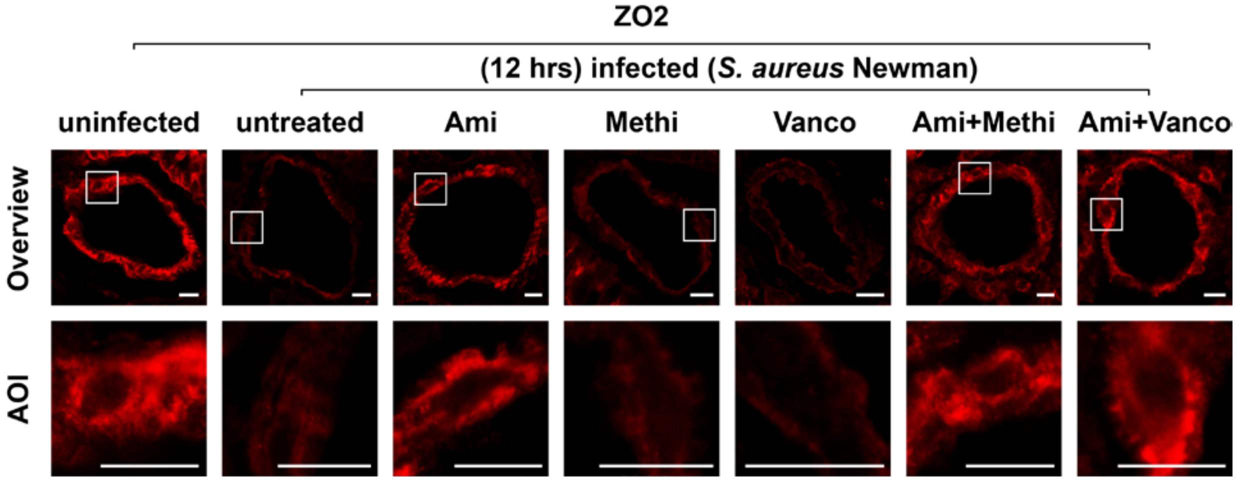
ZO2
(12 hrs) infected (S. aureus Newman)
uninfected
untreated
Ami
Methi
Vanco
Ami+Methi
Ami+Vanco
Overview
AOI

f

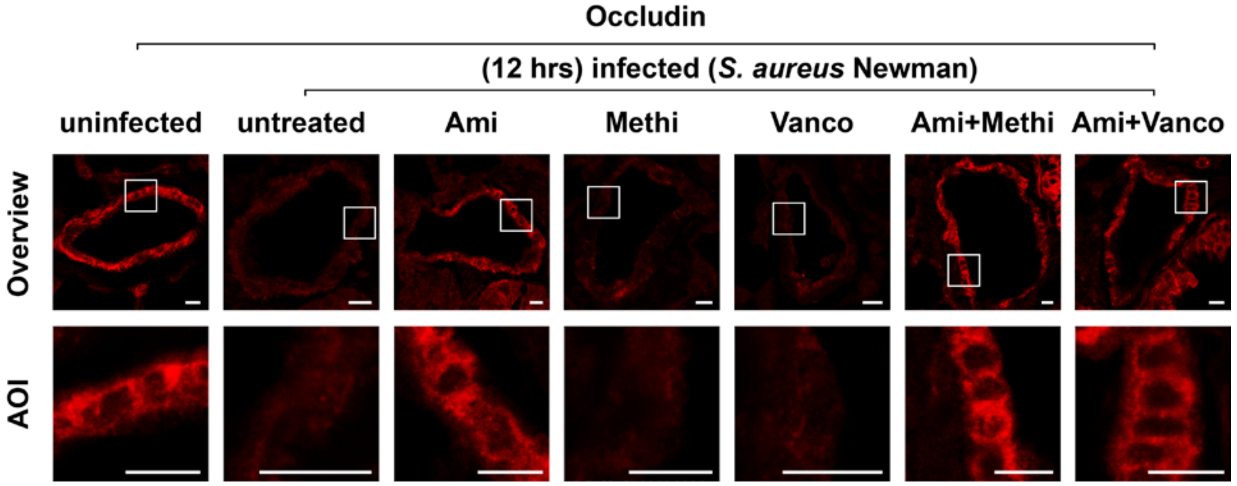
Occludin
(12 hrs) infected (S. aureus Newman)
uninfected
untreated
Ami
Methi
Vanco
Ami+Methi
Ami+Vanco
Overview
AOI

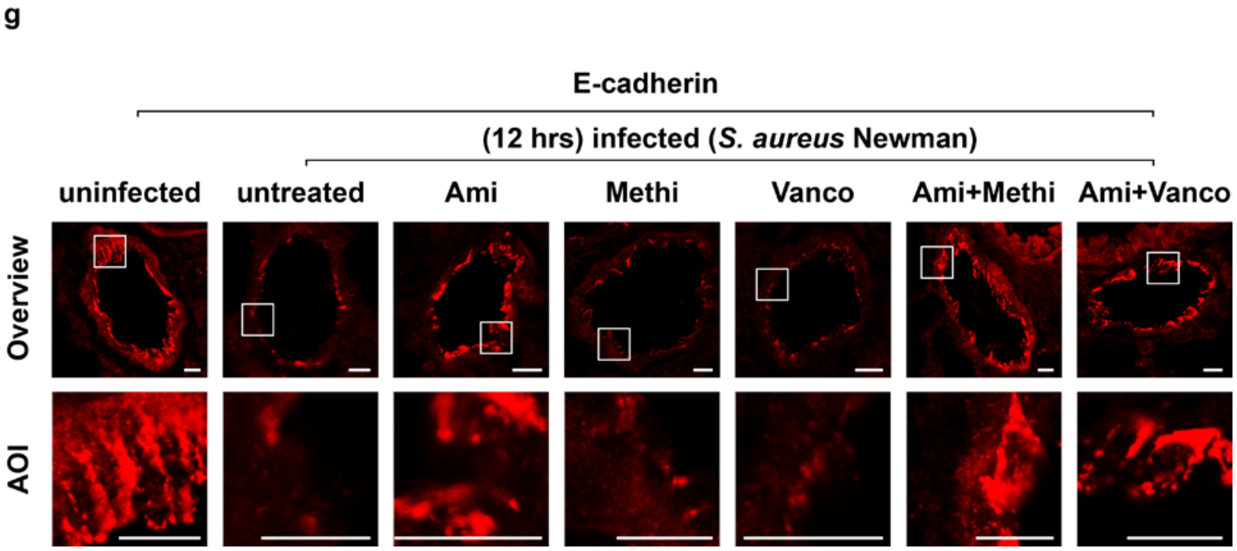

Figure 8. The pharmacological treatment with amitriptyline and antibiotics cures sepsis and lung edema, induced by *S. aureus* strain Newman

Wt mice were infected with *S. aureus* Newman and were either left untreated or treated with amitriptyline (Ami) alone (16 mg/kg, i.p.) 1 hr after infection or treated with amitriptyline combined with either methicillin (Methi) or vancomycin (Vanco) (100 mg/kg each) 1 hr and 9 hrs after infection. Mice were sacrified 12 hrs after infection. Liver and spleen were treated as described above to count colony-forming units (CFUs) **(A)**. Data are presented as mean ± SD of three experiments. *, significant differences between uninfected and infected samples; #, significant differences between treated and untreated samples (all $P<0.05$; *t*-test). Lung sections were obtained and stained with H&E for lung edema **(B)** (scale bar is 100 μm), Cy3-labeled anti-GR1–antibodies for myeloid cell trafficking **(C)** (scale bar is 50 μm) or Cy3-labeled antibodies against ZO1 **(D)**, ZO2 **(E)**, occludin **(F)**, or E-cadherin **(G)** for tight junctions (TJs) and analyzed by light or confocal fluorescence microscopy. The degradation of TJs is shown in the original image and in an area of interest (AOI) (scale bar is 10 μm). All images are representative of three independent experiments.

Supporting Figure 9:

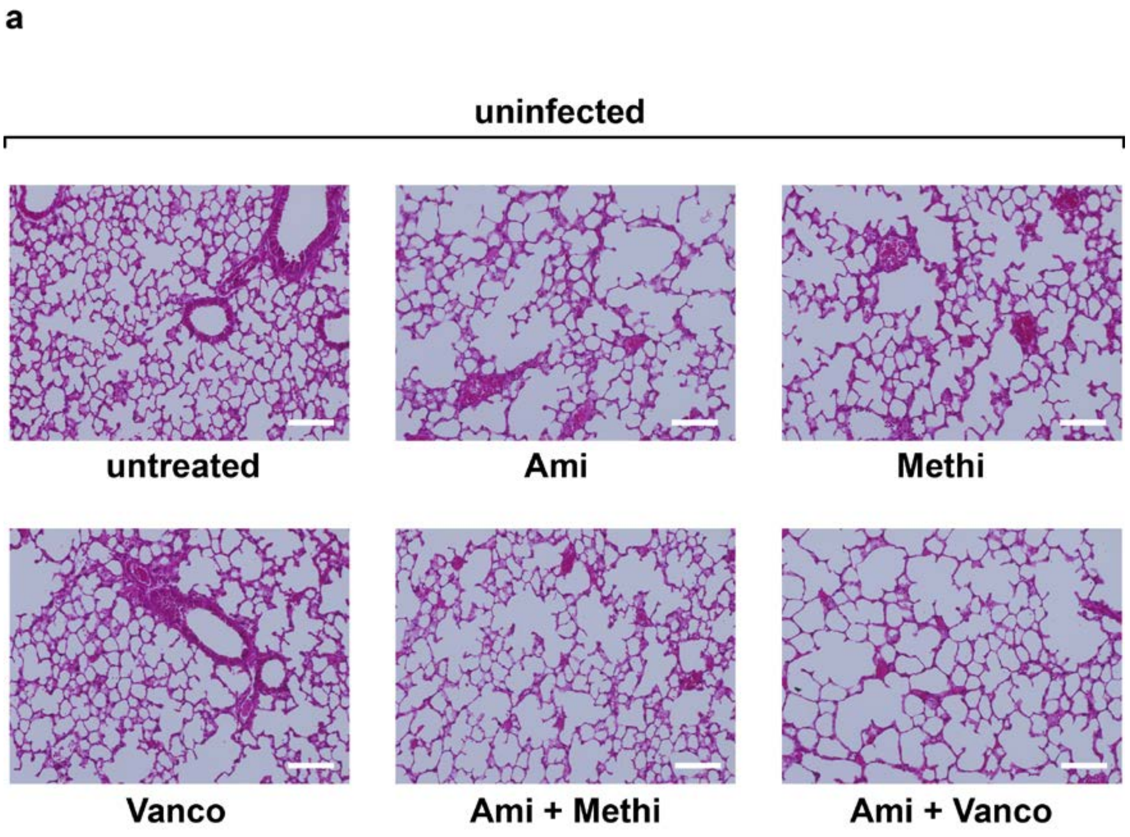

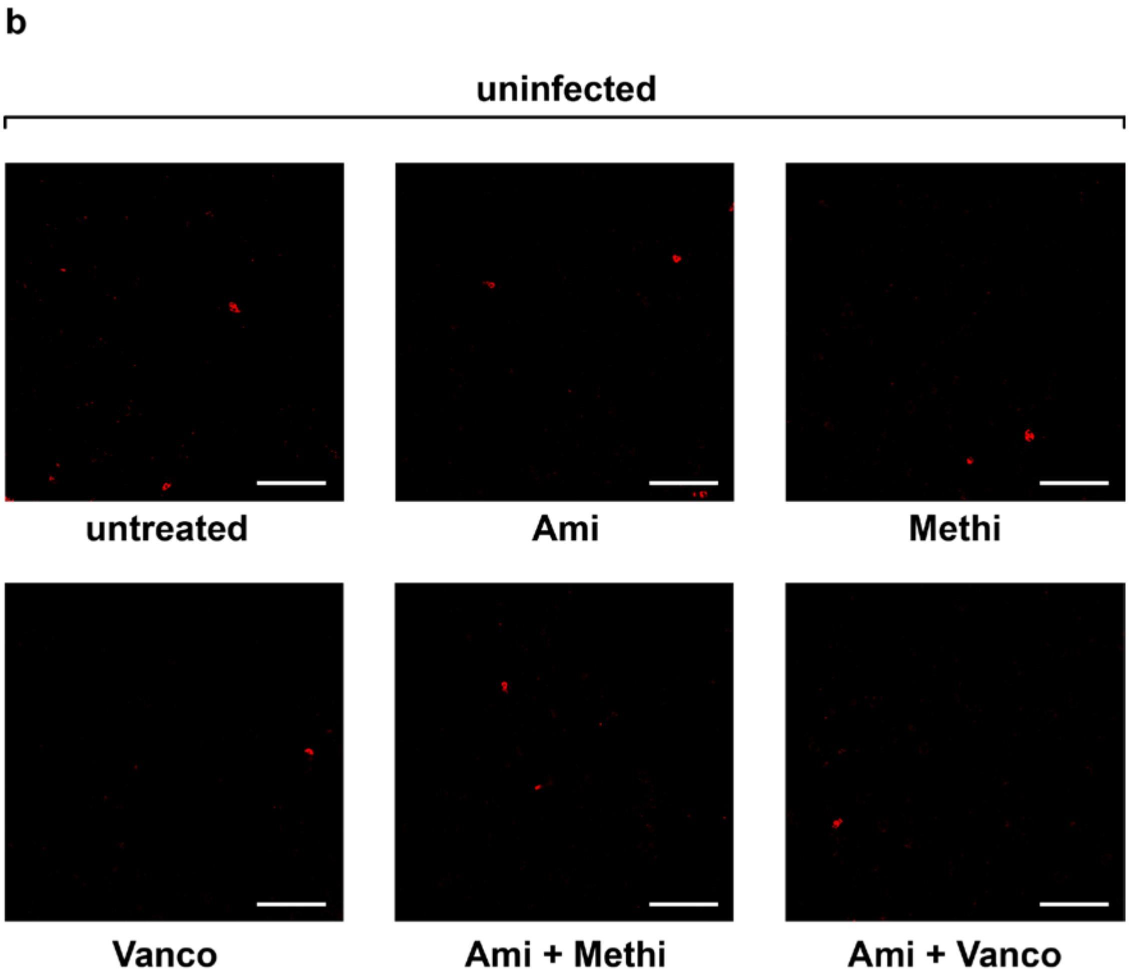

c

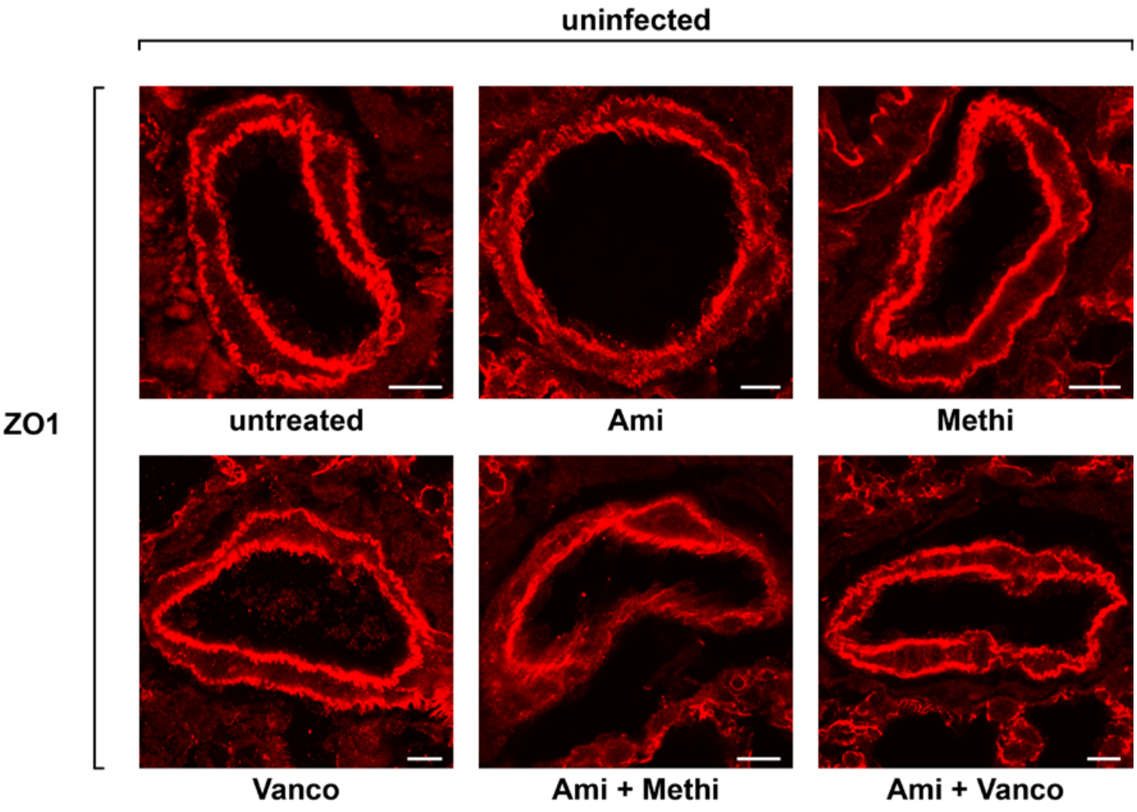
uninfected
ZO1
untreated
Ami
Methi
Vanco
Ami + Methi
Ami + Vanco

d

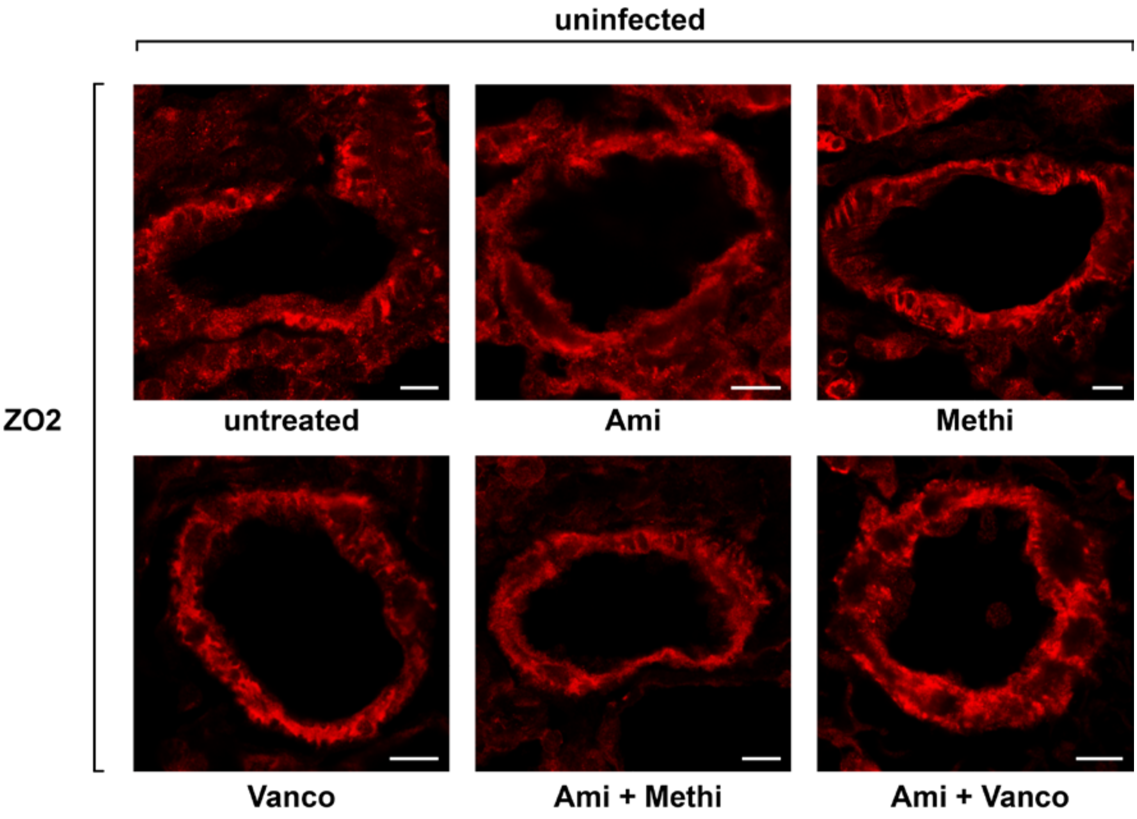
uninfected
ZO2
untreated
Ami
Methi
Vanco
Ami + Methi
Ami + Vanco

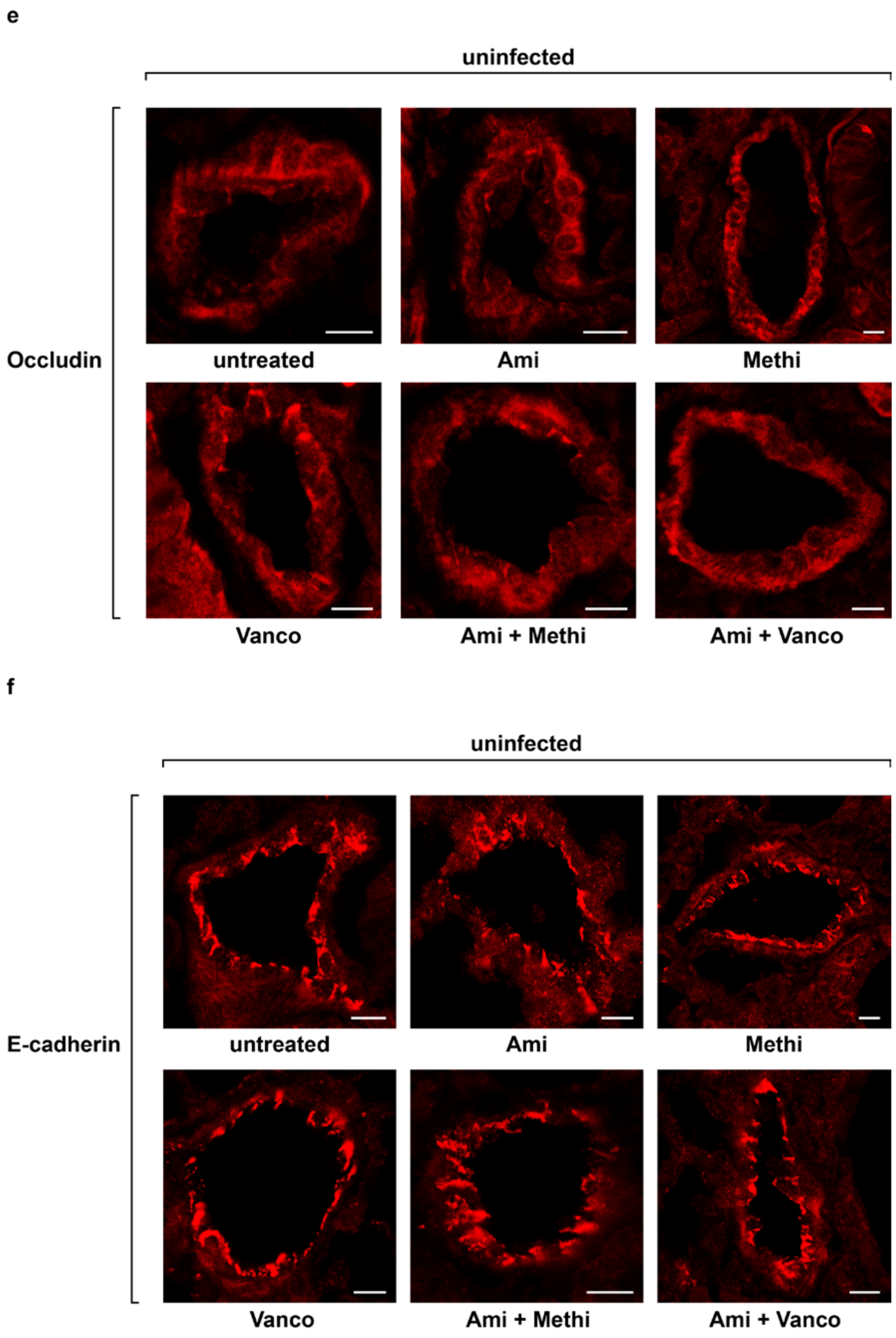

Figure 9. Neither amitriptyline nor antibiotics alter lung morphology, myeloid cell density or tight junctions distribution in uninfected mice

Uninfected wild-type (wt) mice were treated with either amitriptyline (Ami) (16 mg/kg, i.p.), methicillin (Methi) (100 mg/kg) or vancomycin (Vanco) (100 mg/kg) or a combination of amitriptyline with either methicillin or vancomycin for 12 hrs. Three mice per group were sacrificed, lung sections were obtained and stained either with H&E for lung edema **(A)** (scale bar is 100 μm) or with Cy3-labeled anti–GR1-antibody for myeloid cell trafficking **(B)** (scale bar is 50 μm). For detection of tight junction (TJ) proteins, lung sections were stained with Cy3-labeled antibodies against ZO1 **(C)**, ZO2 **(D)**, Occludin **(E)** or E-cadherin **(F)**. Scale bar is 10 μm. Images were obtained by confocal microscopy and are representative of three independent experiments. The original image and an area of interest (AO1) are shown.
